# DNA Damage Signalling and Repair Inhibitors: The Long-Sought-After Achilles’ Heel of Cancer

**DOI:** 10.3390/biom5043204

**Published:** 2015-11-20

**Authors:** Denis Velic, Anthony M. Couturier, Maria Tedim Ferreira, Amélie Rodrigue, Guy G. Poirier, Fabrice Fleury, Jean-Yves Masson

**Affiliations:** 1 Genome Stability Laboratory, CHU de Québec Research Center, HDQ Pavilion, Oncology Axis, 9 McMahon, Québec City, QC G1R-2J6, Canada; E-Mails: velic.d@gmail.com (D.V.); anthony.couturier.1@gmail.com (A.M.C.); Amelie.Rodrigue@crchudequebec.ulaval.ca (A.R.); 2 Department of Molecular Biology, Medical Biochemistry and Pathology, Centre de Recherche sur le Cancer, Laval University, Québec City, QC G1V-0A6, Canada; E-Mails: maria.tedim-ferreira.1@ulaval.ca (M.T.F.); guy.poirier@crchul.ulaval.ca (G.G.P.); 3 DNA repair regulation team, UFIP, CNRS UMR 6286, Université de Nantes, 2 rue de la Houssinière, Nantes 44322, France; E-Mail: fabrice.fleury@univ-nantes; 4 CHU de Québec Research Center, Oncology Axis, 2705 boul. Laurier, Quebec city, QC G1V-4G2, Canada

**Keywords:** DNA repair, inhibitors, ATM, ATR, CHK1, CHK2, MRE11, RAD51, PARP

## Abstract

For decades, radiotherapy and chemotherapy were the two only approaches exploiting DNA repair processes to fight against cancer. Nowadays, cancer therapeutics can be a major challenge when it comes to seeking personalized targeted medicine that is both effective and selective to the malignancy. Over the last decade, the discovery of new targeted therapies against DNA damage signalling and repair has offered the possibility of therapeutic improvements in oncology. In this review, we summarize the current knowledge of DNA damage signalling and repair inhibitors, their molecular and cellular effects, and future therapeutic use.

## 1. Introduction

**Figure 1:**
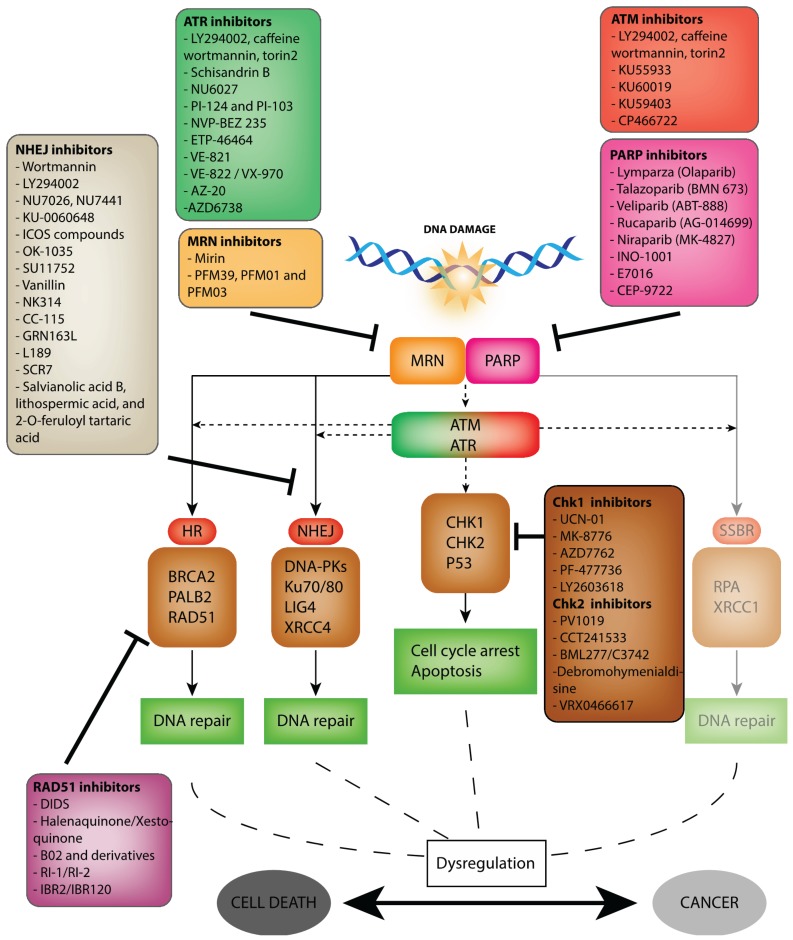
The DNA damage response pathways and its inhibitors.

All living organisms can suffer from deleterious attacks from extrinsic agents as well as intrinsic sources such as reactive oxygen species. One of the most harmful lesions found in genomic DNA are double-strand breaks (DSBs), whose massive cytotoxicity is the basis for conventional DSB-inducing agents, such as ionizing radiation (IR), radiomimetic drugs and topoisomerase II inhibitors, currently used as treatment of choice for cancer therapy. Unfortunately, such treatments come with a lack of tumour specificity, resulting in severe side effects that negatively impact on patient’s life. Current research efforts are now directed toward identifying small inhibitory molecules targeting specific pathways involved in signalling and repairing DSBs. These pathways collectively form a complex network, termed the DNA damage response (DDR), evolved to neutralize DNA lesions and prevent transmission of incorrect genetic information to daughter cells during cell division. In normal cells, the DDR can coordinate cell cycle arrest, DNA repair and apoptosis, and consists of three classes of proteins, each exerting critical functions. Sensors detect the presence of DNA lesions, signal transducers generate and amplify the DNA damage signal, and effectors induce DNA repair, cell cycle delay, programmed cell death or senescence (for review see [1,2]). Cancer cells, for a large part, have compromised DDR pathways, some of them being dependent on a sole back-up pathway for survival. The use of inhibitors against essential components of a back-up pathway, in a synthetic lethal approach, is a promising avenue to specifically eradicate cancer cells. Another inhibitor-based approach to therapy is the potentiation of the effects of DNA-damaging anti-cancer agents, particularly in instances of therapeutic resistance. This review aims to provide a comprehensive view of key inhibitors of DSB DNA damage signalling and repair proteins (outlined in Figure 1).

To respond to DNA damage, cells have implemented the DNA Damage Response. DDR is a signal transduction pathway that implicates different actors depending on the type of damage and/or the cell cycle phase. Firstly, the damage is detected by sensor proteins such as MRN (MRE11-RAD50-NSB1) or PARP1 (Poly(ADP-ribose) Polymerase 1). These sensors recruit the apical kinases Ataxia telangiectasia mutated (ATM) or Rad3-related (ATR). The signal is further amplified and the response results in repair (following diverse mechanisms), cell cycle arrest and/or apoptosis. Single-strand breaks are repaired by Single-Strand Break Repair (SSBR—not discussed in this review). Double-strand breaks are repaired by two main mechanisms: Homologous Recombination (HR) and Non-Homologous End-Joining (NHEJ). HR involves mediators such as BRCA2, RAD51 and PALB2. NHEJ brings into play proteins such as DNA-dependent protein kinase catalytic subunit (DNA-PKcs), Ku70/80, Ligase IV and XRCC4. During DDR pathway, cells require effectors, activated by ATM and ATR, such as CHeckpoint Kinase 1 (CHK1), Checkpoint Kinase 2 (CHK2) or p53 to arrest the cell cycle or enter in apoptosis. A dysregulation of these processes can lead to cell death or, more seriously, to mutagenesis and cancer. When cancer does occur, targeting the principal DDR actors allows the promotion of cell death in order to limit cancer progression. Specifics inhibitors have been designed to target different actors of the DDR pathway. Major inhibitors, discussed in this review, are represented in different boxes in this figure.

## 2. The ATM and ATR Kinases

The ATM (Ataxia-telangiectasia mutated) gene was identified in 1995 from a genetic disorder called ataxia-telangiectasia (A-T) [3]. Ataxia telangiectasia (AT), is a syndrome characterized by neuronal degeneration, immunodeficiency, chromosomal fragility and extreme sensitivity to ionizing radiation [4]. Identified the following year [5], the ataxia and Rad3-related (ATR) gene came to be known for its essentiality for the viability of proliferating human [6] and mouse cells [7,8,9]. When hypomorphically mutated, the gene has been linked to a rare autosomal-recessive disease, the Seckel syndrome, marked by growth and mental retardation, microcephaly, and chromosomal instability [10]. ATM and ATR belong to the phosphatidylinositol 3-kinase-related kinase (PIKK) family of serine/threonine protein kinases, which also includes DNA-PKcs. These kinases are major DDR transducers, which propagate upon DNA damage a message alert by initiating signalling cascades through phosphorylation of numerous targets, including H2AX, p53 and the checkpoint kinases Chk1 and Chk2. To ensure maintenance of genomic integrity, ATM and ATR crucially halt the cell cycle via Chk2 and Chk1, allowing time for repair or invoke cell death mechanisms in the irreparable (for review see [11]). ATM is the primary responder to DSBs, while ATR responds to a variety of lesions including intra-strand crosslinks, oxidative damage, replication blocks, and polymerase toxins [12], with overlapping but non-redundant activities (for review see [13]). The cellular effects of down-regulating ATM and ATR have been the focus of many early studies, using either dominant-negative, small interfering RNA (siRNA) depletion, or chemical inhibition (e.g., with caffeine) approaches [14,15,16,17]. One therapeutically interesting phenotype that emerged from ATM or ATR deficiencies was the increased sensitivity to cell killing by genotoxic agents due to compromised cell cycle checkpoints. This sparked interest in therapeutically using ATM and ATR inhibitors as radio- or chemo-sensitizers, or as a monotherapy in tumours addicted to particular DNA repair pathways. However, the road to finding specific inhibitors of ATM/ATR has been long and winding. One example of this challenge goes as far as 2008. The small molecule CGK-73 was reported to inhibit both ATM and ATR kinases and block checkpoint signalling with great selectivity, but the study was later retracted [18]. Although the long search for selective inhibitors of ATM and ATR has not yet paid off, some of them remain clearly promising. Below is a brief summary of the dual ATM/ATR, ATM or ATR inhibitors (depicted in Figure S1) described to date and the latest advances in the field.

### 2.1. Dual ATM/ATR Inhibitors

#### 2.1.1. Caffeine, Wortmannin and LY294002

The PIKKs ATM and ATR present a high level of sequence similarity and share common targets [19]. These features constitute a difficult challenge for the development of specific inhibitors. Early studies made use of caffeine or wortmannin to block the ATM and ATR kinases. Caffeine and wortmannin exhibited radio- and chemo-sensitizing capabilities but were shown to be non-specific inhibitors that cross-react with phosphatidylinositol 3-kinases (PI3K) and PI3K-related kinases including DNA-PKcs [14,20,21,22]. More recently, Tsabar and colleagues revealed that caffeine impaired resection during DNA break repair by reducing the levels of nucleases Sae2 and Dna2 [23]. Nevertheless, caffeine and wortmannin present a lack of selectivity and high *in vivo* toxicity thus rendering the development of these drugs for the clinic obsolete [24,25].

The first synthetic inhibitor of the PI3K family and PI3K-like family, named LY294002, was characterized at the beginning of the 1990s by the Eli Lilly Company [26]. In this study, LY294002 was used to inhibit PI3K activity in human neutrophils and the proliferation of smooth muscle cells in cultured rabbit aortic segments. Subsequently, LY294002 was shown to be an inhibitor of ATM and ATR [27,28,29,30]. For the next 10 years, several inhibitors were designed from LY294002. Although these show an increased affinity for the PI3K family, there have been reports showing they could act non-specifically by targeting other PI3K-related kinases and proteins apparently unrelated to the PI3K family as well [31,32].

#### 2.1.2. Torin2

Torin2 is a compound developed to overcome the pharmacological limitations of Torin1 (a mTOR selective inhibitor 1) [33]. This compound is also a potent inhibitor of ATR, ATM and DNA-PK in PC3 AktS473D cells [34]. Interestingly, it exhibits an anti-proliferative activity across a panel of cancer cell lines. *In vivo*, Torin2 causes significant growth inhibition of KRAS mutant lung tumours when used in combination with AZD6244 (MEK1/2 inhibitor).

### 2.2. Selective ATM Inhibitors

#### 2.2.1. KU-55933

In 2004, Hickson and KuDOS Pharmaceuticals Ltd discovered the ATM inhibitor KU-55933, which will eventually become one of the most used in laboratories worldwide. KU-55933 has been described as a cell-permeable, potent, selective and ATP-competitive inhibitor of ATM [35]. KU-55933, with an IC50 of 13 nmol/L and a Ki of 2.2 nmol/L *in vitro*, showed a >100-fold higher potency against ATM compared to other PIKK family members, with no selectivity toward a panel of 60 unrelated protein kinases. Lipophilicity issues have, however, limited its potential for clinical use [36,37]. Nonetheless, KU-55933 can induce cell death in senescent breast, lung, and colon carcinoma cells through p21, in an ATM-dependent manner [38]. Using KU-55933, Eaton *et al.* also characterized the role of ATM in the overall regulation of ribonucleotide reductase subunit expression/stability and proper mtDNA copy number dynamics/expression in the presence and absence of induced DNA damage [39]. Recently, KU-55933 has been shown to sensitize several radioresistant cells, such as bladder cancer cells bearing a DAB2IP gene defect [40] and non-small cell lung cancer cells [41]. Consequently, these findings have revived the potential use of KU-55933 in a clinical setting.

#### 2.2.2. KU-60019

In an attempt to improve the specificity of PI3K-like protein inhibitors, KU-60019 was designed by Golding and colleagues [37]. KU-60019 is able to inhibit the DNA damage response, reduce AKT phosphorylation and prosurvival signalling, and effectively radiosensitize human glioma cells. Failure by KU-60019 to reduce AKT phosphorylation and to mediate radiosensitization in A-T fibroblasts, suggested specific targeting of ATM [37]. This drug has similar, if not identical target specificity to KU-55933, with little to no non-specific target effects at 1 μmol/L against a panel of 229 protein kinases. It was also more efficient than KU-55933 at blocking radiation-induced phosphorylation of ATM downstream targets. Studies have demonstrated that KU-60019 radiosensitizes several glioblastoma cell lines [42,43]. Recently, this inhibitor has been shown to be harmful for PTEN mutant cancer cells in tumour xenograft models. This toxicity was reversible by reintroduction of wild-type PTEN [44]. Finally, it has been reported that KU-60019 significantly increases doxorubicin-induced chemosensitization of MCF-7 cells, suppressing their proliferation, supporting the use of KU-60019 as a promising strategy for non-invasive breast cancer [45].

#### 2.2.3. KU-59403

Another ATP competitive inhibitor, KU-59043, was considered as a serious candidate for clinical development, owing to its increased potency, selectivity and solubility, compared to other KU drugs [46]. KU-59403 was shown to be non-cytotoxic in several human cancer cell lines (SW620, LoVo, HCT116, and MDA-MB-231) and was found to have a good tissue distribution and significant chemosensitization without major toxicity. However, KU-59403 has never reached clinical trial steps and no data have been published since.

#### 2.2.4. CP466722

This drug was initially identified in a targeted compound library screen for potential ATM inhibitors, as non-toxic and very specific against inhibition of ATM-dependent phosphorylation events [47]. Rainey and colleagues showed that a transient inhibition of ATM was sufficient to sensitize cells to IR and suggested that CP466722 could be used in a therapeutic perspective. However, a recent study has found that CP466722 is cytotoxic in both MCF-7 and SKBr-3 cell lines by inducing apoptosis [48].

### 2.3. Selective ATR Inhibitors

#### 2.3.1. Schisandrin B

Nhishida *et al.* identified schisandrin B (SchB) as a selective ATR inhibitor by screening herbal extracts and ingredients, although inhibition of ATM was also observed at high concentrations [49]. By focusing on how SchB could be implicated in ATR inhibition, Tatewaki and colleagues found that SchB is a mixture of diastereomers gomisin N (GN) and γ-schisandrin (γ-Sch), in which the former is the active component [50]. More precisely, GN was found to exert its inhibitory action via stereospecific interaction with ATR. SchB can enhance doxorubicin-induced apoptosis of cancer cells but not normal cells [51], prevent doxorubicin-induced chronic cardiotoxicity and enhance its anticancer activity *in vivo* [52]. Recently, SchB has been implicated as an anti-UVB-induced damage agent in HaCat cells [53]. While its role as an ATR inhibitor is promising, further studies are needed to validate SchB as a sensitizing agent for anti-cancer therapy.

#### 2.3.2. NU6027

NU6027 is a potent inhibitor of ATR activity in several breast and ovarian cancer cell lines, but its initial discovery as a CDK2 inhibitor renders it less interesting [54]. Still, NU6027 reduces G2/M arrest following DNA damage, decreases RAD51 focus formation and increases cytotoxicity of the major classes of DNA-damaging anticancer agents, but not the antimitotic agent paclitaxel. Furthermore, A2780 cells with functional p53 and mismatch repair (MMR) are sensitive to a combination of NU6027 and cisplatin treatment, whereas p53 mutant cells with functional MMR are susceptible to temozolomide (TMZ) combined with NU6027. Importantly, NU6027 is synthetically lethal when DNA single-strand break repair is impaired, either through poly(ADP-ribose) polymerase inhibition or defects in XRCC1. Recently, Sultana *et al.*, confirmed that ATR inhibition by NU6027 leads to synthetic lethality in XRCC1-deficient ovarian cancer cells [55]. The resulting enhanced cytotoxicity was accompanied by accumulation of double-strand DNA breaks, G2/M cell cycle arrest and increased apoptosis.

#### 2.3.3. PI-124 and PI-103

In a screen for PI3K inhibitors, PI-124 and PI-103 were identified as being potent inhibitors of ATR, but also of mTOR protein kinases [56]. Recently, the combination of olaparib and PI-103 has been shown to enhance radiation-induced death in triple-negative breast cancer (TNBC) cells and to significantly reduce tumour volume in a mouse xenograft model. PI-103 inhibitor treatment was accompanied by persistent γ-H2AX foci, indicating delayed repair of DNA strand breaks (associated with decreased RAD51 and p-DNA-PK, but not ATR). Remarkably, PI-103 alone increased poly(ADP-ribose) and phosphorylated extracellular signal-regulated kinase levels, while downregulating BRCA1 [57]. Consequently, the combination of PI3K signalling pathway and PARP inhibitions could be a suitable approach to enhance effects of radiation in BRCA-proficient TNBC.

#### 2.3.4. NVP-BEZ235

Identified from a high throughput screening (HTS), NVP-BEZ235 possesses significant inhibitory activity against ATR kinase [58]. However, it also presents affinity for mTOR and DNA-PKcs proteins, which makes it a less specific compound [59]. As a result, research is now focused on how NVP-BEZ235 can be used to block the PI3K/Akt/mTOR pathway in bladder cancer [60], malignant pleural mesothelioma [61] or in endometrial carcinoma [62]. Particularly, NVP-BEZ235 is a potent radiosensitizing compound for Ras-overexpressing tumours [63]. This drug is now in phase 2 of clinical trials for its PI3K/mTOR inhibitory function.

#### 2.3.5. ETP-46464

In addition to NVP-BEZ 235, Toledo *et al.* studied the action of ETP-46464 and showed that it possesses an ATR inhibitory activity, but lacked selectivity since it could inhibit the activities of PI3Kα, mTOR and DNA-PKcs [58]. ETP-46464 was found to destabilize replication-stalled forks, suppress the ionizing radiation-induced G2/M checkpoint and enhance the presence of micronuclei or fragmented nuclei in cells. Furthermore, ETP-46464 was specifically toxic for p53-deficient cells (the overexpression of cyclin E enhances this effect). Unfortunately, ETP-46464 seems to have poor pharmacological properties in mice, which limits its use for therapy.

#### 2.3.6. VE-821

An HTS held by Vertex Pharmaceuticals identified VE-821 as a potent and selective ATR inhibitor [64]. ATR inhibition by VE-821 blocks downstream Chk1 activation and enhances DNA damage mediated by cisplatin or gemcitabine. Of interest, VE-821 presents low activity against ATM, DNA-PK and a large panel of unrelated protein kinases [65]. VE-821 has been shown by different groups to IR sensitize 12 human cancer cell lines [66], pancreatic cancer cells [67] and ovarian cancer cells [68]. Accordingly, ATR inhibition by VE-821 leads to the inhibition of radiation-induced G2/M arrest in cancer cells.

#### 2.3.7. VE-822/VX-970

Fokas *et al.* identified this analogue of VE-821 as a powerful ATR inhibitor that radiosensitized pancreatic cancer cells [69]. Importantly, VE-822 was well tolerated in mice and did not enhance toxicity in normal cells and tissues [69]. VE-822 was the first selective ATR inhibitor to enter clinical development and is now known as VX-970. This inhibitor was tested in a series of *in vitro* and *in vivo* lung cancer models. VX-970 was shown to block ATR activity in tumours and dramatically enhance the efficacy of cisplatin across a panel of patient-derived primary lung xenografts [70]. These data suggest that VX-970 may have the potential to increase the efficacy of DNA damaging therapy in patients with lung cancer. A phase I clinical trial to assess the safety, tolerability and pharmacokinetics of VX-970 in combination with cytotoxic chemotherapy is currently ongoing (ClinicalTrials.gov: NCT02157792). To a lesser extent, VE-822 has been shown to suppress the activation of NF-κB and increase E1-dependent replication of the HPV16 genome [71].

#### 2.3.8. AZ-20

Belonging to the sulfonylmorpholinopyrimidines family, AZ-20 was synthetized by the AstraZeneca (London, UK) plc group from the optimization of a high quality screening hit and showed a selective affinity for ATR [72]. It compellingly inhibits the growth of LoVo colorectal adenocarcinoma tumour cells *in vitro* and has high symptom-free exposure in mice following moderate oral doses.

#### 2.3.9. AZD6738

AZD6738, developed by AstraZeneca in 2012, is an analogue of AZ-20, a potent and selective ATR inhibitor [36]. No data have yet been published about this inhibitor, but it is one of the few that has been tested in a completed clinical trial (NCT01955668). A recent plenary session from Glenn Clack (AstraZeneca) also described two clinical studies with AZD6738 that will test the monotherapy and combination therapy hypotheses, along with an overview of the supporting pre-clinical *in vitro* and *in vivo* data [73] (NCT02223923).

## 3. The Chk1 Kinase

### 3.1. Roles of Chk1 in the DNA Damage Response

Checkpoint kinase 1, commonly named Chk1 or Check1, is a serine-threonine kinase. Chk1 is encoded by the *CHK1* (CHEK1) gene and is located on chromosome 11 (11q22-23) in humans. Chk1 is highly conserved from budding yeast (Rad27) to humans, particularly its amino-terminal catalytic domain [74]. Chk1 has a C-terminal regulatory SQ/TQ domain which may be involved in its translocation between the cytoplasm and the nuclear compartment. Chk1 is one of the key proteins in cell cycle control which activates cell cycle checkpoint initiation. Chk1 is thus involved in several steps of the cell cycle from S to G2/M phase. Chk1 is also a cell regulator and a DNA damage sensor. In response to DNA damage, Chk1 phosphorylates and thus inhibits Cdc25 phosphatases, particularly Cdc25A which is its main target. This causes Cdc25 degradation, inhibition of cyclin-Cdk and prevention of mitotic phase entry [75]. Activation of Chk1 results in the initiation of cell cycle checkpoints, cell cycle arrest, DNA repair and cell death to prevent damaged cells from progressing through the cell cycle. Therefore, Chk1 is crucial in genomic stability but can also be a source of resistance to anticancer therapies.

### 3.2. Phosphorylation of Chk1

Chk1 activation occurs through ATR-mediated phosphorylation of two conserved sites, Ser317 and Ser345 [76]. These phosphorylation events promote the autophosphorylation of Chk1 on Ser296, which triggers its cell cycle arrest and DNA repair functions [77,78]. Chk1 pSer345 site has been described as an important phosphorylation site for its catalytic activation and is considered as a marker of DNA damage [79]. These post-translational modifications trigger the release of Chk1 from the chromatin fraction into the soluble compartments [80] which allows cell cycle arrest and repair processes. Released and activated Chk1 then phosphorylates a number of downstream targets to control cell cycle transition and DNA damage repair [81]. DNA DSB repair proteins involved in homologous recombination, such as the key protein RAD51, have been identified among the targeted proteins [82]. Indeed Chk1 is involved in the activation of DNA repair by homologous recombination [83]. In response to DNA lesions, Chk1 physically interacts with and phosphorylates RAD51 on Thr309, promoting the recruitment of RAD51 at DNA damage sites [82]. Treatment with Chk1 inhibitors impairs HU-induced RAD51 foci formation, confirming the essential role of Chk1 as activator of HR [82].

### 3.3. Targeting Chk1 as a Novel Strategy in Cancer Therapy

Chk1 plays a key role in regulating replication checkpoints and the DDR since this kinase is mainly responsible for G2/M checkpoint. The main checkpoint regulator in G1/S phase is p53 and in numerous cancers (about 50% of cases) p53 is either mutated or null. Given that most tumour cells suffer defects in the p53 pathway, cancer cells have to maintain functional S and G2/M phases mediated by Chk1 to avoid premature mitotic entry which could lead to mitotic catastrophe. This latter status is characterized by phosphorylation of Histone H3 (on Ser10) and aberrant mitotic spindle formation [84]. These p53-related defects have been taken advantage of to kill cancer cells and it has been shown that using Chk1 siRNA enables to enhance apoptosis in p53-deficient tumour cell lines [85,86]. P53 inhibitors are described in [87].

In addition, a high level of Chk1 is often described in various types of cancers, including breast cancer, pancreatic cancer, non-small cell lung carcinoma and leukemia. In one report, lung cancer cells expressing high levels of Chk1 were hypersensitive to Chk1 inhibitors. Recently, Sarmento *et al.* have shown that Chk1 overexpression in T-cell acute lymphoblastic leukemia (T-ALL) enables cellular proliferation and survival by preventing replication stress [88]. Chk1 overexpression contributes to cancer resistance by delaying mitotic entry and thereby it has been associated with tumour grade and poor prognosis [89,90]. Chk1 contributes to cell survival by inducing G2 arrest and signalling to the HR pathway for DNA repair [82].

### 3.4. Inhibitors of Chk1

In the last decade, increasing publications describe the potential of targeting Chk1 with small molecule inhibitors (depicted in Figure S2) as a new direction for anticancer therapy [91]. A number of Chk1 inhibitors have been developed against different cancer types and are under clinical investigation [92,93,94]. The main inhibitors of Chk1 activity are described below.

#### 3.4.1. UCN-01 (7-Hydroxystaurosporine)

This inhibitor is one of the first molecules directed against Chk1 [95]. It has frequently been used in *in vitro* experiments but its nonselective kinase inhibition and its lack of clinical efficacy have not allowed its progression beyond phase II of clinical trials [96,97].

#### 3.4.2. MK-8776 Inhibitor (Previously Known as SCH900776) (Pyrazolo [1,5-*a*] Pyrimidine Derivative)

MK-8776 is a potent and selective Chk1 inhibitor in clinical development. Guri *et al.* have developed a functional approach to identify the most potent Chk1 inhibitor, the mechanism-based phenotypic screening. This method is based on the assessment of γ-H2AX foci accumulation, known as a marker of DSBs and collapsed replication forks. The Chk1 inhibitor SCH900776 was thus discovered [98]. This molecule is potent and highly reactive. It induced γ-H2AX accumulation and suppressed pS296 Chk1 autophosphorylation within two hours. This Chk1 inhibitor has been combined with gemcitabine treatment and has shown chemotherapy sensitization by increasing the level of double-stranded DNA damage. The efficiency of Chk1 inhibition was demonstrated by the increase and persistence of γ-H2AX foci. The *ex vivo* assay of intracellular γ-H2AX foci in gemtacibine-exposed cells from patient serum was performed by flow cytometry measurement and confirmed this efficiency [92]. Another study has shown the ability of MK-8776 to sensitize pancreatic cancer cells in which HR is proficient to gemcitabine-radiation treatment [99]. In this study, treatment of pancreatic cancer cells with the Chk1 inhibitor promoted a sensitization to gemcitabine-radiation that is associated with inhibition of RAD51 foci formation. This result confirmed the role of Chk1 in the recruitment of RAD51 on DNA damage sites. In non-small cell lung cancer cells similar observations were made after using MK-8776 in combination with pemetrexed, a classic anti-metabolite drug. The authors concluded that the levels of Chk1 may be a predictive marker for MK-8776 sensitivity [100]. These findings have allowed the MK-8776 molecule to enter in phase II clinical trials in combination with chemotherapy.

#### 3.4.3. AZD7762

AZD7762 is an ATP competitive Chk1 inhibitor and is equally efficient against Chk2. Since HR allows the restart of replication forks in treated cancer cells, its inhibition has been proposed as a novel strategy for cancer therapy [101]. Inhibition of HR repair and DNA damage-induced cell cycle checkpoint response by Chk1 inhibition led several scientific teams to test other inhibitors such as AZD7762 for sensitizing different cancers to conventional therapy. It has been described that lung cancer cells expressing high levels of Chk1 were hypersensitive to AZD7762. This suggests a correlation between Chk1 inhibitor-mediated sensitivity and elevated amounts of Chk1 [89]. In one report, the combination of AZD7762 with gemcitabine and ionizing radiation was assessed *in vitro* and allowed sensitizing pancreatic cells to radiation. In addition to abrogating the G2/M checkpoint, the inhibition of HR was suggested as the main mechanism for this sensitization [83]. More recently, AZD7762-mediated sensitization has been shown to be more selective and efficient against pancreatic cancer stem cells, known to be refractory to standard chemo- and radiotherapy [102,103]. The *in vitro* cytotoxicity of AZD7762 has also been investigated on MM cell lines. Combined with alkyling agents, such as melphalan, AZD7762 promoted apoptosis and mitotic catastrophe of p53-mutated MM cells [104].

#### 3.4.4. PF-477736

PF-477736 is a selective and competitive inhibitor for the Chk1 ATP site. Its specificity is one hundred times stronger for Chk1 than for Chk2. Ovarian cancer often responds well to treatment with PF-477736 but it rapidly generates metastasic and chemoresistant forms [105]. A subtype of ovarian cancer, high-grade serous (HGS) carcinoma, is characterized by a defect in DNA repair including mainly the HR pathway [106]. The treatment of HGS cells by PF-477736 combined with topotecan (topoisomerase I inhibitor) improved and synergized the cellular toxicity by inducing apoptosis [105]. Sarmento *et al.* demonstrated that treatment of T-ALL cells by PF-477736 promotes apoptotic cell death. PF-477736-induced Chk1 inhibition led to impaired replication combined with the abrogation of G2/M checkpoint in T-ALL cells. Interestingly, this inhibitor did not significantly affect normal thymocyte cells *in vitro* which shows the ability of PF-477736 to discriminate normal and cancer cells [88].

#### 3.4.5. LY2603618

This molecule is a potent and selective inhibitor of Chk1 and was selected after high throughput screening. Like most of the other inhibitors, LY2603618 binds to the ATP binding site of Chk1. The phenotype of LY2603618-treated cells was similar to Chk1-depleted cells by siRNA, proving that the Chk1 inhibition pathway is involved. In a recent preclinical study, the characterization of LY2603618 has been reported [107]. By inhibiting Chk1, this molecule was able to abrogate the G2/M DNA damage checkpoint in colon cancer cells treated with gemcitabine and doxorubicine. It has been demonstrated that LY2603618 potentiated the effect of DNA damage drugs *in vitro*. This result was also validated *in vivo* using a tumour xenograft approach. LY2603618 is the most advanced molecule in phase II trial evaluation and a number of studies using combinations with other DNA damage drugs are ongoing [108].

#### 3.4.6. Other Inhibitors

The interest of inhibiting Chk1-mediated DNA damage checkpoints in cancer has been demonstrated in numerous studies. Therefore the pharmaceutical industry is interested in the development of new more specific and more efficient molecules [77]. Candidates such as XL844 and CHR-124 have been proposed but the clinical phase of their development has been discontinued [109]. The development of novel Chk1 inhibitors for anticancer therapies based on specific checkpoint defects may lead to personalized cancer treatment.

## 4. The Chk2 Kinase

The serine/threonine protein kinase Chk2 has been firstly described as the mammalian ortholog of *Saccharomyces cerevisiae* Rad53 [110]. Chk2 bears three important domains: a SQ/TQ cluster domain (SCD), a forkhead-associated (FHA) domain located at the N-terminus and a kinase domain near the C-terminus of the protein [111]. In instances of DSBs, Chk2 is thought to be the primary effector kinase to be activated and to spread the DNA damage signal to downstream effectors of the DDR. Its activation is initiated by ATM phosphorylation on Thr68 and other residues in its N-terminus, which triggers conformational changes and auto-phosphorylation on many residues for complete activation [111,112,113]. Chk2 activity is mediated by many proteins such as 53BP1, MDC1, NBS1 [114,115,116,117,118]. The main role of activated Chk2 is to block the cell cycle progression by inactivating phosphorylation of Cdc25 family proteins (A, B and C) [119]. Chk2 also targets proteins involved in DNA repair, p53 signalling, and apoptosis. Among these, Chk2 phosphorylates BRCA2 on Thr3387, which is critical for RAD51 localization to DSBs [120]. The kinase also facilitates HR through BRCA1 phosphorylation on Ser988 [121]. Chk2 inhibitors (depicted in Figure S2) have been envisioned as attractive therapeutic agents for their potential at sensitizing cancer cells to DNA-damaging agents and their ability to protect normal cells against DNA damage-induced apoptosis [93,122,123,124]. To date, five main molecules targeting Chk2 in cells have shown interesting results, all of them being ATP-competitive inhibitors.

### 4.1. PV1019 ([7-Nitro-1H-Indole-2-Carboxylic Acid {(4-[1-(Guanidinohydrazone)-Ethyl]-Phenyl}-Amide]

When a high-throughput screen for Chk2 inhibitors was conducted on a 100,000-compounds library, using an immobilized metal ion affinity-based fluorescence polarization assay (IMAP), a member of the guanidylhydrazones, named NSC109555, emerged as the sole hit [125]. NSC109555 exhibited highly selective inhibition against Chk2 kinase activity on histone H1, but was not cell penetrant [126]. Removal of the second guanidylhydrazone group and replacement of one side of the aryl urea with a 7-nitroindole motif linked through an amide gave rise to a new cell-penetrant molecule, PV1019, with improved affinity and conserved selectivity for Chk2 [127]. Cellular evidence for the inhibition of Chk2 kinase activity was confirmed by assessment of Chk2 autophosphorylation, and phosphorylation of downstream targets Cdc25c and HDMX. Other analogues of PV1019 with increased cell permeability and selectivity have been developed [122]. PV1019 is the only guanidylhydrazone tested in cells to be able to decrease IR-mediated apoptosis in mouse thymocytes [127]. Another potential therapeutic value highlighted by this study was that PV1019 potentiated effect of two topoisomerase I inhibitors, topotecan and camptothecin, or IR-treatment in various cancer cell lines, including the human ovarian cancer cell lines OVCAR-4 and 5, and the brain glioma cell line U251. In U251 cells, the potentiation of IR by PV1019 (5 µM) was enhanced 1.4-fold, which represents the only case of radiosensitization of a cancer cell line caused by a selective Chk2 inhibitor.

### 4.2. CCT241533

In a kinome profiling study, Caldwell and colleagues discovered one compound belonging to the 2-(quinazolin-2-yl)-phenols molecules that inhibited Chk2 activity [128]. Optimization of the hit led to CCT241533 synthesis, a more efficient Chk2 inhibitor which interacts with Met304 and Asn352 of Chk2 via hydrogen bonds. CCT241533 had a high passive permeability and inhibited Chk2 in cancer cells while showing no effect in combination with DNA-damaging agents, such as SN38, gemcitabine, mitomycin C, bleomycin or etoposide on p53-deficient cells HT29 and HeLa, but interestingly potentiated effect of two PARP inhibitors AG14447 and olaparib [129]. This was shown by decreased cell growth in both HT29 and HeLa cancer cell lines in the presence of 1.5 µM and 3 µM CCT241533 respectively. Furthermore, combination of CCT241533 and olaparib enhanced apoptosis in HeLa cells by potentiating PARP inhibitor cytotoxicity in a Chk2-dependent manner. The cytotoxicity of the combination of CCT241533 and olaparib was attributed to the Chk2-mediated inhibition of BRCA1 phosphorylation and concomitant HR impairment.

### 4.3. 2-(4-(4-Chlorophenoxy)Phenyl)-1H-Benzimidazole-5-Carboxamide Hydrate**/**BML277/C3742

The first selective Chk2 inhibitor, which belongs to the benzimidazole class of inhibitors, was obtained through a high-throughput screening of a 2-arylbenzamidazole library for binding to purified Chk2, followed by Structure-Activity Relationship (SAR) study to improve specificity of the first hits [130]. It has been shown that this compound binds Chk2 via three main interactions: (1) a hydrogen interaction between the benzimidazole H1 and the carbonyl group of Glu302 and the amide NH of Met304; (2) binding of the carboxamide group with many residues in the pocket of the kinase and (3) interaction of the 4-chlorophenyl substituent with hydrophobic residues located at the entrance of the ATP-pocket (Leu226, Leu236, Lys245, Leu303 and Glu305) [131]. This molecule showed protective effect in p53 wild-type CD4+ and CD8+ T-cells exposed to IR in a concentration-dependent manner [130]. No study on the cellular effects of this molecule has been published recently.

### 4.4. Debromohymenialdisine and Analogues

Based on the Chk2 inhibitor debromohymenialdisine identified in marine sponge, analogue development led to the synthesis of a derivative compound by substitution of the pyrrole of the hymenialdisine core with an indole group [132,133,134]. This compound showed an increased selectivity and affinity for Chk2 compared to the initial debromohymenialdisine. IR-treatment of non-malignant-cells revealed a protective effect on apoptosis by the molecule [135]. Other analogues were developed based on the substitution of the 2-pyrrole moiety with phenyl rings, increasing selectivity for Chk2 but decreasing its inhibitory effect on Chk2 [136]. No further investigation has been reported as yet with this compound or analogues.

### 4.5. VRX0466617

VRX0466617 was synthesized using a structure-based drug design strategy led by Valeant Pharmaceuticals International starting from an isothiazole carboxamide identified by screening [137]. VRX0466617 exhibits higher affinity and selectivity for Chk2 (IC_50_ = 120 nM, K_i_ = 11 nM) compared to Chk1 (IC_50_ > 10 µM) [138]. This compound showed an effect on IR-treated cells by blocking the kinase activity of Chk2. Phosphorylation on Ser19 and Ser33-35 was inhibited in the presence of the molecule, but the ATM-dependent Thr68 phosphorylation of Chk2 was spared. HDMX is a negative regulator of p53 that is phosphorylated by Chk2 on Ser342 and Ser367 upon exposure to IR, leading to its degradation. VRX0466617 prevented IR-induced HDMX degradation in a concentration-dependent manner. It was found to inhibit IR-induced apoptosis, but did not potentiate effect of DNA-damaging drugs cisplatin and doxorubicin in MCF-7 cancer cell line, nor taxol in BJ-hTERT cells.

Other small molecules, such as UCN-01 [139], AZD7762 [140], or XL844 [141], have been identified to inhibiting Chk2 but these are thought to exhibit equal or higher potency for Chk1 than for Chk2.

In contrast to Chk1 inhibitors, Chk2 inhibitors have not reached the clinical trial stage. Although the experimental setting (e.g., the genetic background of the cancer cell lines and the DNA-damaging agents used) may account for this, there have been conflicting studies as to whether pharmacological inhibition of Chk2 can synergize DNA-damaging agents. A lack of clarity regarding the role of CHK2 inhibition and the context for Chk2 selectivity works also against the favor of Chk2 inhibitors.

## 5. The Poly(ADP) Ribose Polymerase

Poly (adenosine diphosphate [ADP]) ribose polymerase (PARP) inhibitors are a class of drugs which have recently had an investigational growth in the cancer treatment setting due to the increasing understanding of the functions of PARP enzymes in DNA repair. PARP1 and PARP2 are the best studied enzymes out of a 17 PARP protein family [142,143]. Activation of PARP upon DNA damage leads to the synthesis of poly(ADP)-ribose (pADPr), which is one of the earliest steps of DNA damage recognition and signalling in mammalian cells [144]. PARP1 catalysis the addition of pADPr to chromatin-related proteins, increasing the accessibility of repair factors to DNA lesions. Numerous molecules are recruited at DNA-damage sites in a pADPr-dependent manner [142,143,145].

PARP inhibitors are analogues of the nicotinamide moiety of the NAD^+^ (nicotinamide adenine dinucleotide) [146,147,148] and their mechanistic effect is to compete for the substrate binding site of PARP, acting as an effective catalytic (CAT) inhibitor [146,147,149,150]. Inhibiting PARP catalysis blocks base excision repair (BER) and leads to the accumulation of unrepaired single-strand DNA breaks (SSBs), which develop into DSBs in replicating cells. Such DSBs require competent HR repair to allow cell survival [151,152].

The *BRCA1* and *BRCA2* genes code for proteins that have been very well documented for being key for a functional HR repair pathway. Normal cells of BRCA1/2 mutation carriers have one copy of BRCA1 or BRCA2 (BRCA1/2+/−), which allows them to retain their capability to undergo repair with satisfactory proficiency. However, when the wild-type BRCA is lost, these cells become defective in repairing DSBs by HR. Instead, these lesions are repaired by the error-prone mechanisms of NHEJ leading to genomic instability and predisposition to cancer [151,152,153]. Therefore, much of the development of PARP inhibitors has been focused on targeting this weakness of cancers associated with mutations in these breast cancer-related tumour suppressor genes. Accordingly, cells containing a mutation in either of these genes, when treated with PARPi will fail to undergo normal HR repair. This selective targeting of BRCA-deficient cancer cells by PARPi introduces the concept of synthetic lethality, whereby a cell harbouring one of two gene or protein defects is viable while a cell containing both defects is nonviable. Moreover, PARP has been suggested to participate in the restoration of stalled replication forks [152,154] by accumulating at the damaged site and recruiting MRE11 to catalyse DNA-end processing. Therefore by inhibiting PARP, stalled replication forks will not progress and the consequent accumulation of toxic DNA structures will lead to cytotoxicity in HR-deficient cells [155].

Since the discovery of such concept, PARPi have been developed with a highly efficacious CAT inhibitory concentration, at an impressive nanomolar range. Olaparib (AZD-2281; AstraZeneca, London, UK) was the first PARPi to demonstrate clinical activity in *BRCA* mutation-associated cancers. The trial demonstrated that more than 90% of PARP enzymatic activity could be inhibited with no increased toxicity [156]. Following clinical trials also provided proof of concept of olaparib single-agent activity of PARP inhibition in patients with tumours that have genetic loss of function of BRCA1-associated or BRCA2-associated DNA repair [157,158]. Subsequent work has shown that, in addition to BRCA1/BRCA2 mutations, BRCA-like tumours may also have implications for PARPi-based therapy. These tumours have molecular and clinical characteristics in common with tumours occurring in patients with germ line BRCA mutations (gBRCAm) [159]. Furthermore, a wider subgroup of solid tumours that may have deficiency in other HR genes may also cause cancer cells to become sensitive to PARP inhibitors [160,161,162,163]. The clinical success of olaparib and the discovery of tumours with molecular and clinical features similar to gBRCAm-associated tumours, prompted the clinical development of various PARP inhibitors either as single agents or as part of combined treatment with DNA damaging agents in phase I and II clinical trials.

There are at least 8 PARP inhibitors (depicted in Figure S3) that have reached clinical development in oncology [164,165,166]. Lynparza (Olaparib) is a potent (PARP-1 IC_50_ = 5nM) oral PARP1/2 inhibitor [156] whose therapeutic efficacy has recently led to the approval by the US FDA and the European commission (report on the FDA website) for the treatment of patients with platinum-sensitive, recurrent, high-grade serous ovarian cancer with BRCA1 or BRCA2 mutations. This is the first approval of a drug in the PARP inhibitor class and efforts are being made for its potential approval in other types of cancer. An ongoing phase II, TOPARP-A (NCT01682772), is now evaluating olaparib for its anti-tumour activity in metastatic castration resistant prostate cancer, in order to identify molecular signatures of tumour cells in responding and non-responding patients, and to identify predictive biomarkers of olaparib response. Moreover, an ongoing phase II (NCT01078662) study of olaparib monotherapy in BRCA1/2 mutation carriers of different tumour types revealed clinical benefit in prostate and pancreatic cancer as well as activity in ovarian and breast cancer. The gBRCAm-associated pancreatic cancer patients yielded a clinical benefit rate of 57% [167]. Prolonged responses to olaparib across all tumour types observed in this study support the hypothesis that therapy directed against a genetically-defined target has activity regardless of the anatomic organ of origin. Olaparib has been shown to have therapeutic effects in cell lines with mutations in other HR genes such as tumour suppressor PTEN [168]. Such event was later supported by subsequent studies in xenografts of PTEN-deficient endometrial cancer [169,170]. These studies provided a rationale for further studies in whether PTEN loss can serve as a potential biomarker for PARPi sensitivity [171,172,173]. Also, most recently olaparib was suggested to be an attractive therapeutic option as a monotherapy for acute myeloid leukemia patients not suitable for conventional chemotherapy (CT), also giving strong evidence of possible biomarkers to predict PARPi resistance or response [174].

### 5.1. Talazoparib (BMN 673; BioMarin Pharmaceutical Inc., Novato, CA, USA)

*Talazoparib* is an oral PARP1/2 inhibitor with greater *in vitro* activity than any other PARP inhibitor currently in development (PARP-1 IC_50_ = 0.6 nM) [173]. Much like olaparib, BMN 673 has been shown to cause single-agent synthetic lethality in BRCA1/2- and PTEN-deficient cell lines, with potent antitumour activity in animal models of tumours harbouring mutations in DNA repair pathways [173,174,175]. It is especially showing promise as single-agent treatment in patients with advanced ovarian and breast cancer harbouring deleterious BRCA1/2 mutations. Furthermore, BMN 673 has also demonstrated to potentiate the antitumour effects of TMZ, cisplatin (CIS), and carboplatin [173]. These therapeutic effects were achieved with tolerable toxicity, evidence of pADPr inhibition *in vivo* in animal tumour models, and favourable pharmacokinetic properties that allow once a day oral dosing in human patients. It is currently under trial as single agent and in combination therapy with chemo and radiotherapy (RT) in a plethora of different types of cancer such as advanced solid malignancies and deleterious BRCA1/2 mutation-associated cancers (clinicaltrials.gov).

### 5.2. Veliparib (ABT-888; AbbVie Inc., North Chicago, IL, USA)

*Veliparib* is a potent oral PARP-1/2 inhibitor (PARP-1 K_i_ = 5nM) with nanomolar cell-based activity, decent oral bioavailability [176] and apparent CNS penetration [164]. Preclinical studies in breast cancer, melanoma and glioma models demonstrated that veliparib potentiates the CT effect of a number of agents including TMZ, platinum, and irinotecan (CPT), as well as radiation [177]. Veliparib also has the capacity to synergise with olaparib in combination with ionizing radiation in cell line modes of head and neck cancer and lymphoma [178,179]. An ongoing phase I/II (NCT01489865) study is testing the efficacy of a new combination of drugs, with veliparib and a platinum agent, mFOLFOX-6 (modified 5-fluorouracil and oxaliplatin), for patients with metastatic pancreatic cancer (MPC). The combination has demonstrated promising efficacy in MPC, particularly in patients with BRCA2 gene mutations [180]. Other phase I and II trials are exploring the combination with RT and CT, respectively, in various cancer types [181,182].

### 5.3. Rucaparib (AG-014699; Clovis Oncology, Boulder, CO, USA)

*Rucaparib* is an intravenous (IV) PARP inhibitor characterized as a potent PARPi (PARP-1 IC50 = 2 nM) in cell based assays of radio and chemosensitization and is a very potent enzyme inhibitor [183,184,185]. As a solo agent, rucaparib has been demonstrated to sensitize human cancer cells or xenograft tumours with mutated or epigenetically silenced BRCA1/2, indicating a potential role for PARP inhibitors in sporadic human cancers [186]. This inhibitor is mainly being trialled as a monotherapy for different types of ovarian cancer (clinicaltrials.gov). Two ongoing clinical trials, known as ARIEL2 (NCT01891344) and ARIEL3 (NCT01968213) are aiming to evaluate biomarkers to predict how a woman with ovarian cancer may respond to rucaparib and to explore the effect of rucaparib in women with high-grade serous platinum-sensitive relapsed ovarian cancer or endometrioid epithelial ovarian, fallopian tube, or primary peritoneal cancer, respectively. A first time study on leukemic cells using a combined therapy that associates the conventional CT agent fluorouracil (5FU), used as a source of DNA damage, and rucaparib, demonstrated the efficacy and the specificity of this combined therapy in killing both acute myeloid leukemia and acute lymphoid leukemia cells *in vitro* and *in vivo*. This study therefore suggests a new therapeutic strategy able to enhance the cytotoxic effect of DNA-damaging agents in leukemia cells via inhibiting the repair of damaged DNA [187].

### 5.4. Niraparib (MK-4827; Tesaro Inc., Waltham, MA, USA)

*Niraparib* is an orally available PARPi, which demonstrates potent and selective PARP-1 and PARP-2 inhibitory activity (IC_50_ = 3.8 and 2.1 nM respectively) [188,189]. It was firstly studied in patients with BRCA1/2 mutations and sporadic cancers associated with HR repair defects. These demonstrated niraparib to be and to have promising antitumour activity in both BRCA-deficient and sporadic cancers [190]. Niraparib was also found to cooperate with radiation and enhance cell death of neuroblastoma cells *in vitro*. When experimented *in vivo* the radiation combination with niraparib decreased tumour burden and prolonged survival in a metastatic neuroblastoma model. Overall, this study proposed this therapeutic combination to be a promising new therapy for treatment of metastatic neuroblastoma [191]. Currently, this inhibitor is enrolled in a phase I (NCT02044120) trial in a combination regimen with TMZ in recurrent Ewing sarcoma [182]. It is also enrolled in a phase II (NCT02354586) and III (NCT01905592) trial studies as maintenance therapy in ovarian cancer and as a treatment for breast cancer.

### 5.5. INO-1001 (Selleck Chemicals, Euromedex, France)

*INO-1001* is an IV PARPi that shows capability to restore sensitivity to TMZ in a mismatch-repair (MMR) deficient xenograft model of malignant melanoma [192]. This inhibitor has also demonstrated potential in enhancing radiation sensitivity without associated cytotoxicity in mammalian cells [193]. MMR loss of function could potentially be a predictive biomarker of PARPi responsiveness in patients with metastatic melanoma. A most recent IB trial concluded that the administration of INO-1001 in combination with TMZ is safe for metastatic melanoma individuals [194]. No clinical trials are ongoing for this PARPi.

### 5.6. E7016 (Eisai Co., Ltd; Tokyo, Japan)

*E7016* is an orally bioavailable PARP inhibitor that can enhance tumour radiosensitivity and also provide a greater than an additive effect when used in combination with TMZ and radiation [195]. It is currently enrolled in an ongoing phase II (NCT01605162) trial which is exploring its efficacy in combination with TMZ in patients with malignant melanoma [196].

### 5.7. CEP-9722 (Cephalon, Inc.; Frazer, PA, USA)

*CEP-9722* is an oral PARP-1/2 inhibitor and a pro-drug of CEP-8983. Pre-clinical studies [197] demonstrated its capability of sensitizing different types of tumour cells to TMZ, CPT and radiation. Concluded phase I studies have evaluated CEP-9722 maximum tolerated dose (MTD) in patients with advanced or metastatic solid tumours, its antitumour activity as a single agent, its MTD in combination with gemcitabine and CIS in advanced solid tumours and mantle cell lymphoma (NCT01311713), and its safety, pharmacokinetics, and pharmacodynamics as single-agent therapy and as combination therapy with TMZ in patients with advanced solid tumours (NCT00920595). Clinical trials for this inhibitor have been completed or terminated and no other studies are ongoing.

Overall, the utility of PARP inhibitors as therapeutic candidates is not limited to their efficacy in treating malignancies. They also have emerged with a spectrum of actions that incorporates treating other conditions such as cardiovascular diseases, stroke, metabolic disorders, diabetes, and autoimmune disorders [198,199,200,201].

### 5.8. PARP Trapping: Mechanism of Action of PARP Inhibitors

As previously mentioned, many clinically investigated PARP inhibitors, including talazoparib, niraparib, olaparib, rucaparib and veliparib, are highly efficacious at inhibiting PARP CAT activity, with very similar half-maximal inhibitor concentration values in the single-digit nanomolar range. This antitumour activity was thought to be exerted solely by the inhibition of PARP1 and PARP2 CAT activity. However, several studies [202,203,204] have demonstrated that, in addition to CAT inhibition, PARP inhibitors have the capacity of trapping PARP1 and PARP2 at SSB sites, possibly via a poisonous allosteric effect that affects the conformational flexibility and dynamics of PARP, enhancing its affinity for the SSB repair intermediate [202,203]. Trapped PARP-DNA complexes prevent DNA replication and transcription. Thus, these DNA-PARP complexes kill cancer cells more effectively than genetic depletion of PARP [202,205]. This recent mechanism of action is denominated PARP poisoning or trapping.

If CAT inhibition was the only way of antitumour activity, one would expect that similar IC_50_ values of CAT activity would result in similar PARPi cytotoxicity potency. Whereas, it has been demonstrated that the ability of PARP inhibitors both in terms of single-agent activity in BRCA-deficient cells and in the sensitization of cancer cells to alkylating agents, differ by several orders of magnitude. Rucaparib, olaparib, and niraparib are comparable regarding cancer cytotoxicity, whist talazoparib is approximately 25- to 100-fold more potent. On the other hand, veliparib is ~1000- to 10,000-fold less potent than talazoparib in cytotoxicity assays [202,203,206]. This magnitude of differences in cytotoxicity cannot only be explained by differences in CAT inhibition or by differential off-target activity of the PARP inhibitors. In fact, olaparib and veliparib have similar selectivity profiles against 13 of the 17 human PARP family members and do not demonstrate off-target activity [207]. Talazoparib is also comparable to both these PARPi in its selectivity profile [204], with its non-off-target activity most recently demonstrated by the resistance effect PARP1 knock out cells acquired to talazoparib [203]. Likewise, cells with PARP1 loss-of-function mutations are 100-fold more resistant to olaparib than wild-type cells [208]. These data indicate that PARP1 protein is the critical mediator of the cytotoxic effect of PARP inhibitors, arguing against the suggestion that these differences in cytotoxicity result from off-target activities.

As mentioned above, the proposed mechanistic effect that could be behind such differences is PARP trapping, where PARP inhibitors induce an allosteric conformational change in PARP1 and PARP2, stabilizing their associations with DNA and therefore forming lethal PARP-DNA complexes. Direct evidence has been provided, demonstrating that in fact, PARP inhibitors exert their cytotoxicity primarily by this trapping mechanism rather than by classic enzyme inhibition and that this capacity varies widely across different PARP inhibitors [202,203]. Five PARPi have been extensively tested in different cell lines for their trapping capacity [202,203]. The stabilization of the PARP-DNA complexes by PARPi is reversible and these complexes have been demonstrated both in intact cells and in cell-free systems [202,203,209]. Veliparib is primarily a CAT inhibitor with little trapping activity, consistent with the effect observed in PARP1 loss-of-function mutant cell lines showing resistance to veliparib only at very high drug concentrations [202]. On the other hand, olaparib, niraparib, and rucaparib trap PARP ~100-fold more efficiently than veliparib. Talazoparib is the most potent PARP trapper studied to date, promoting PARP-DNA complexes ~100-fold more efficiently than olaparib, niraparib, and rucaparib [202,203]. Overall, these data suggest in fact that the anticancer cytotoxicities of PARP inhibitors do not have a linear relationship with CAT inhibition, but instead correlate very well with trapping potency. The clinical efficacy data for PARP inhibitors in cancer patients with deleterious BRCA gene mutations are consistent with the *in vitro* cytotoxicity levels of the PARP inhibitors, nicely correlating the clinical activity with the ability to trap PARP [204].

Moreover, attempts have been made to decipher the differences in the trapping potency of equally strong PARP1 CAT inhibitors [202]. Studies suggest that the level of inhibitor-induced reverse-allosteric-effect may be correlated with the size or bulk of PARP inhibitors, which may ultimately be related to the degree of PARP trapping. It is understood, by a substantial body of structural data, that PARP inhibitors do in fact differ considerably in overall size/scaffold and consequently in binding modes. Comparing to the bulkier PARP inhibitors, such as olaparib, niraparib, rucaparib and talazoparib, veliparib is the smallest amongst them [202,203], and demonstrates to be the least potent in trapping PARP. Accordingly, it also does not appear to induce conformational changes on PARP1 in microsecond molecular dynamic simulations studies [210]. On the other hand, olaparib demonstrates to cause CAT domain steric bumps on the HD subdomain. These steric bumps trigger conformational rearrangements in the DNA-binding domains and stabilize the PARP1-DNA complex, therefore implicating the HD subdomain as a crucial structural element in the proposed reverse-allosteric mechanism [210]. The fact that veliparib does not induce steric bumps can be explained by its methylpyrrolidine moiety being too small to make significant contacts with the CAT domain [176,210]. Further supporting this relationship of inhibitor size and degree of PARP trapping, a potent CAT inhibitor in pre-clinical development similar to veliparib in size and binding mode, also shows low levels of PARP trapping activity [210,211]. However, among the bulky PARP1 inhibitors, no correlation can be firmly established. For instance, talazoparib has been shown to be the most potent PARP-trapping inhibitor tested to date, despite its small size compared to olaparib [203]. Thus, the molecular size of the inhibitor alone, seems insufficient to justify these vast differences between trapping potency. Recent studies suggest that the shape and flexibility of inhibitors may contribute to the efficiency of trapping PARP-DNA complexes [203], by dictating the extent and type of binding interactions. Also, inhibitors with noticeable ability to stabilize the PARP-DNA complexes appear to interact with the D-loop, localized at the outer border of the NAD^+^ site. For instance, rucaparib and niraparib appear to bind close to the D-loop, and olaparib forms a hydrogen bond with the D-loop residue Met890 [210]. Talazoparib has unique tripartite chemical scaffolds that are rigid and stereospecific [173,212]. The stereospecific 4-fluorophenylsubstituent is favourably oriented to interact with the Met890 backbone via a water molecule and the Tyr889 side chain on the D-loop. This Tyr889 residue, can assume multiple side-chain conformations [213,214], however binding of a rigid stereospecific inhibitor such as talazoparib may sterically restrict the side-chain flexibility. These interactions suggest that the degree of PARP-trapping capacity may be associated with the extent to which the inhibitor interacts with the D-loop residues. These residues are structurally linked to the HD subdomain. Therefore, structural changes in this domain will consequently influence the conformational flexibility of the CAT domain. Additional studies are required to elucidate the structural consequences of the decreased side-chain flexibility of the D-loop residues, such as Tyr889, on PARP trapping [215].

Overall, the effectiveness in PARP trapping may be an important factor to consider when formulating a therapeutic regimen involving a PARP inhibitor (single agent or in combination), therefore it is of highly importance to elucidate the molecular basis that explains the differential PARP trapping between the inhibitors. Newly available structural data on the non-catalytic domains [216,217], combined with co-crystal structures of the inhibitor-bound PARP1 CAT domain is a major step into answering such question [147,163,218,219].

## 6. The MRE11-RAD50-NBS1 Complex

**Figure 2:**
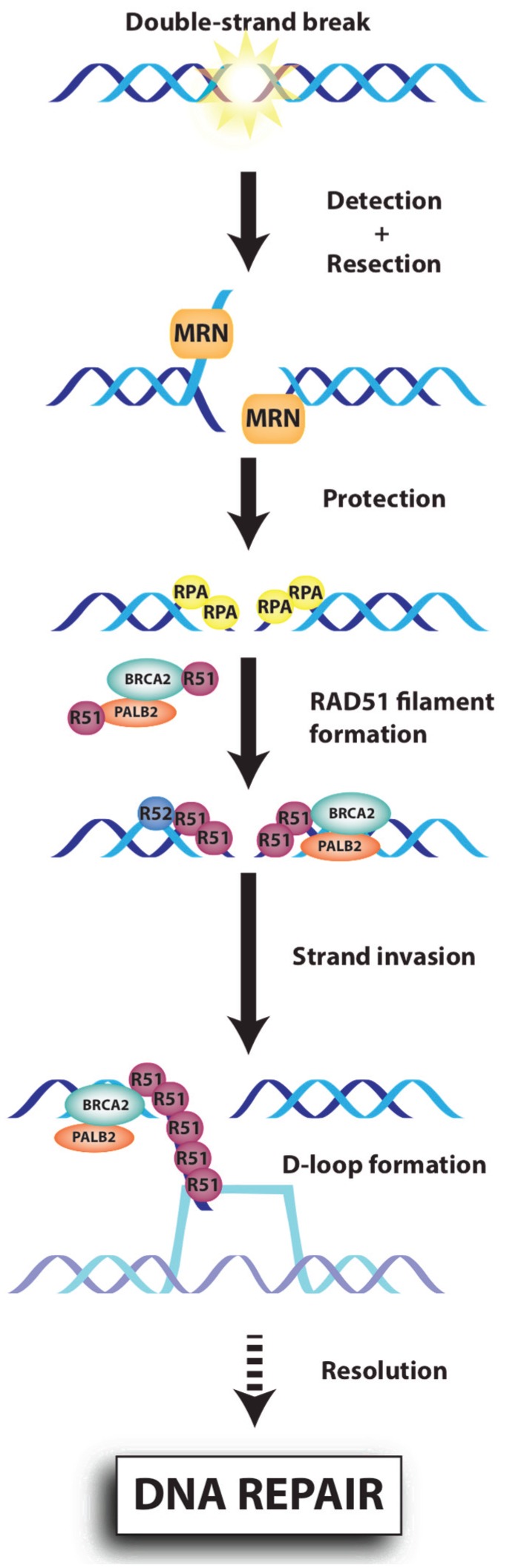
Schematic representation of DNA repair by homologous recombination. During Homologous Recombination (HR), the double-strand break is recognized by the MRE11-RAD50-NBS1 (MRN) (MRE11-RAD50-NBS1) complex, which resects DNA (with EXO1, CtIP and BLM) to create a 3'-overhang DNA that will be protected by RPA. The BRCA1-PALB2-BRCA2 complex then mediates the replacement of RPA by RAD51. RAD51 nucleoprotein filament, invades the complementary DNA template (a step called strand invasion), leading to the formation of the Displacement-loop (D-loop) structure. Following branch migration and resolution, faithful DNA repair occurs.

The MRE11-RAD50-NBS1 (MRN) complex (Figure 2) acts both as a sensor and an effector in the DDR, with crucial roles in the detection, signalling and repair of DSBs. Mre11 and Rad50 were originally characterized in genetic screens from *Saccharomyces* *cerevisiae*, the Mre11 mutant being defective in meiotic recombination [175] and Rad50 mutant sensitive to DNA damage [176]. NBS1 was later isolated as a member of the ternary complex, binding MRE11 and RAD50. Mutations in *NBS1* gene are linked to Nijmegen Breakage Syndrome, an autosomal recessive disorder characterized by high cancer incidence, cell cycle checkpoints defects and IR sensitivity [177]. In human, mutations in MRE11 cause A-T like disorder [178] and a RAD50 deficiency has been reported in NBS-like disorder [179]. Null-mutations in each of these three genes cause embryonic lethality in mice [180,181,182]. MRE11 acts as a dimer [183], binds DNA [184,185], presents exo- and endonuclease activities [186,187] and has the ability to synapse DNA ends [183]. RAD50 carries an ATPase activity used to maintain DNA ends in close proximity [188]. NBS1 recruits DNA repair and checkpoint proteins at the DSB site via phospho-dependent interactions, namely with γ-H2AX, checkpoint adaptor MDC1 and HR regulator CtIP (CtBP-interacting protein) [189,190,191,192]. MRN has been extensively studied for its essential role in the resection of DSBs that it owes to the endo- and exonuclease activities of MRE11. Resection contributes to the generation of 3' single-stranded DNA tails, which are essential for ATM stimulation and subsequent cell cycle checkpoint activation as well as for initiation of HR repair [193,194]. Moreover, recent studies have revealed that resection plays a critical role in the DSB pathway choice by committing cells to HR while suppressing NHEJ [195,196]. As dysfunction in the MRN functionality leads to hypersensitivity to DNA-damage agents by impairment of ATM pathway, inhibiting one of the MRN complex components represents an attractive strategy to induce cancer cell death in combination with DNA-damaging treatment. In an effort to identified small molecules inhibiting the MRN complex, a few MRE11 inhibitors (depicted in Figure S4) have been discovered.

### 6.1. Mirin

Mirin, the first MRE11 inhibitor described, was isolated from a screen of a 10,000 small-molecule library (DIVERSet, Chembridge Corporation), wherein compounds were tested for their inhibitory effect on the phosphorylation of a peptide derived from γ-H2AX by ATM [197]. With the most efficient inhibitory effect (IC_50_ = 66 µM), mirin appeared to target the MRN complex rather than ATM. More specifically, mirin was found to block the MRN-dependent activation of ATM without affecting the kinase activity and to abolish MRE11 nuclease activity (100 µM). Exposing mammalian cells to mirin led to the abrogation of the IR-induced G2/M checkpoint and failure of HR repair. Mirin was later shown to down-regulate NHEJ repair efficiency as well [198]. The broad range of cellular effects associated with mirin denoted a lack of HR specificity that has limited the enthusiasm for its potential clinical utility. To date, no clinical study has been initiated with mirin.

### 6.2. PFM39, PFM01 and PFM03

Recently, novel small molecules have been discovered to inhibit endo- or exonuclease activity of MRE11 [196]. A library of molecules, derived from mirin with substituents in the styryl moiety and replacement of the pseudothiohydantoin ring by rodanine moiety, was created. The mechanism of action of mirin was first studied by co-crystallization of Mre11 from *Thermotoga maritima* with mirin. It appeared that the His61 (corresponds to His63 in human MRE11) and Asn93 residue may be involved in the interaction with mirin. The His61 residue controls dsDNA end accessibility to the active site and Asn93 may interact with single-strand DNA. Based on this and the structure of mirin, three molecules where developed, namely PMF39, PFM01 and PFM03. PFM39, which has an amino group substituting the hydroxyl group, binds between the His61 loop and the adjacent loop (Asn93-Lys97) and inhibits the exonuclease activity of MRE11, as mirin does. PFM01, which has a rodanine ring plus an isobutyl chain on the nitrogen, and PFM03, which has a sec-butyl chain, may bind near the dimer interface of MRE11 to inhibit its endonuclease activity. The three PFM molecules induced a decrease in MRE11-dependent DSB resection that was supported by reduced ssDNA formation, as detected using an antibody against BrdU, and impaired IR-induced RAD51 foci in G2 cells. Mirin and PFM39 cause a G2 repair defect 8 h after IR treatment in HR deficient cells while PFM01 and PFM03 have no effect, highlighting the fact that exo- and endonuclease inhibitors confer different DSB repair phenotypes. PFM01 and PFM03 enhanced NHEJ and reduced HR while PFM39 inhibited HR but have no effect on NHEJ. No translational applications have been led yet with these molecules, but because of the importance of DNA resection in DNA repair and the fact that these small molecules influence the DNA repair pathway choice, it will be interesting to evaluate their combined impact in a synthetic lethal strategy.

## 7. The RAD51 Protein, a Central HR Protein

RAD51 is an evolutionarily conserved recombinase that is central to DSB repair by HR (Figure 2) and crucial for cell survival, as knock-out of the *Rad51* gene in the mouse led to an early embryonic death [199]. With the assistance of HR mediator proteins, such as BRCA2, PALB2, and the RAD51 paralogs, RAD51 is recruited to the DSB sites and polymerizes onto the resection-generated ssDNA ends, forming a nucleoprotein filament that promotes strand invasion and exchange between homologous DNA sequences [200,201,202,203]. The complete RAD51 crystal structure has not been determined yet, but analysis of the crystallized BRC-RAD51 fusion protein, NMR and mutations analysis of RAD51 revealed that RAD51 holds two domains: a N-terminal part that binds both ssDNA and dsDNA and a C-terminal part that binds ATP [204,205]. *In vitro*, RAD51 recombinase activity is manifested by DNA binding, homologous pairing, strand exchange and DNA-dependent ATPase activity [200]. *In vivo*, it takes the form of discrete nuclear foci at the DNA break sites [206]. It has been suggested that RAD51 regulation is important for cell homeostasis, as a variety of cancers, such as leukemia [207], pancreatic [208], breast [209], prostate [210] and non-small lung cancers [211], have been linked to an overexpression or up-regulation of RAD51 while others, including breast and colorectal cancers, exhibit RAD51 underexpression [212]. Overexpression of RAD51 has been shown to stimulate HR efficiency and promote chemotherapy resistance [212,213]. Previous experiments with siRNA against RAD51 have highlighted the potential of RAD51 down-regulation at increasing radio- or chemosensitivity of cancer cells [214,215]. In this light, RAD51 has become a recognized target to anti-cancer therapies and finding molecules (these inhibitors are depicted in Figure S5) that target either its expression level, recombinase activity, or protein interactions is the current focus of many research studies.

### 7.1. DIDS

DIDS (4'-Diisothiocyanostilbene-2,2'-disulfonic acid) has been previously known for its ability to inhibit chloride channels [216]. By screening a library of 185 molecules, Ishida *et al*. [217], have found that DIDS is able to inhibit RAD51 strand-exchange activity. Further *in vitro* studies revealed that DIDS inhibits both the ssDNA and dsDNA-binding activity of RAD51, binds directly to RAD51 and prevents DNA invasion and D-loop formation by RAD51. Recently, Lamont *et al.* [218], have shown that DIDS provides an antileukemic effect by targeting RAD51. In normal B cells, expression of activation-induced cytidine deaminase (AID) by antigenic stimulation leads to immunoglobulin switch class, by creating double-strand breaks. Evidences showed that attenuating HR sensitizes AID-expressing neoplastic B cells to AID-mediated cytotoxicity [219]. In their study, Lamont *et al.*, showed that DIDS inhibits RAD51 foci formation and prevents malignant B cells to use the HR pathway to repair AID-induced DSB. The mechanism of action of DIDS on RAD51 is still unknown, but it is suggested that the molecule may bind near the DNA-binding domains of RAD51, preventing the recombinase from binding DNA [218].

### 7.2. Halenaquinone/Xestoquinone

Takaku *et al.* used 160 crude extract fractions from marine sponges in a D-loop formation assay screening [220]. They highlighted the ability of the compound halenaquinone to significantly decrease RAD51 DNA-invasion and strand-exchange activities. Interestingly, a derivative molecule from halenaquinone, xestoquinone, that lacked the oxygen at position C-3 had no effect on RAD51 DNA pairing. The authors also showed by Surface Plasmonic Resonance that halenaquinone directly binds to RAD51, with a higher affinity than xestoquinone. *In vitro*, halenaquinone was found to inhibit RAD51 binding to dsDNA but not to ssDNA. In cells, the compound had a dose-dependent effect on RAD51 foci formation after IR treatment, but not on RAD51 expression, suggesting that it interfered with RAD51 retention at DSB sites. Xestoquinone had no effect on RAD51-dsDNA binding or foci formation, suggesting that the oxygen at position C-3 is important for halenaquinone activity on RAD51. No other investigation has been yet published on halenaquinone inhibitory effect.

### 7.3. B02 and Derivatives

Huang *et al.* [221] developed a high-throughput method based on FRET (Fluorescence Resonance Energy Transfer) to screen a 200,000 molecules library from the NIH repository and identify inhibitors of RAD51 strand-exchange activity. From the 17 compounds identified, four molecules emerged as the most potent RAD51 inhibitors, as determined by D-loop assays, and were tested for selectivity towards RAD51. B02 (3-(Phenylmethyl)-2-[(1E)-2-(3-pyridinyl)ethenyl]-4(3H)-quinazolinone**)** was found the most efficient inhibitor of RAD51 activity (IC_50_ = 27,4 µM), with no effect on RecA, the *E. coli* homolog of RAD51, and structurally unrelated RAD54. B02 derivatives were then synthetized to develop RAD51 inhibitors of higher efficiency. It revealed that B02-3a and B02-3b, which contain an ethyl and a m-methylphenyl group, respectively, instead of a benzyl group, are at least as efficient as B02 on RAD51 D-loop formation and strand-exchange activities.

Three studies were then carried out to assess the effect of B02 in a cellular environment. A first study showed that B02 was also able to decrease RAD51 foci formation by 3.8-fold (50 µM) and HR efficiency by 8-fold in HEK cells after IR treatment [222]. The authors revealed that B02 sensitized RAD51 siRNA-treated HEK293 cells to cisplatin and MEF cells to a combined MMS and PARP1 inhibitor (AZD2281) treatment. Huang and collaborators [223] tested the effect of B02 in combination with different chemotherapeutics agents, such as etoposide, cisplatin, doxorubicin, and topotecan, on the MDA-MB-231 human breast cancer cell line. B02 was able to sensitize MDA-MB-231 cells to all drug combinations, the most important effect of B02 being seen with cisplatin. They also highlighted the inhibitory effect of B02 combined with cisplatin treatment in mouse xenografts by studying MDA-MB-231 proliferation. B02 was able to disrupt RAD51 foci formation in MDA-MB-231 cells in mouse xenografts. Another study led by Alagpulinsa *et al.* [224] investigated the effect of B02 on multiple myeloma (MM) cells combined with doxorubicin treatment. Alone, doxorubicin treatment induced RAD51 foci formation in multiple myeloma cells, whereas pre-treatment with B02 blocked doxorubicin-induced RAD51 foci formation and increased the γ-H2AX foci number, due to unrepaired DSB caused by the doxorubicin treatment. Using HR assays, they estimated that B02 inhibits HR at least 6-fold.

### 7.4. RI-1 and RI-2

By screening a library of 10,000 molecules DIVERSet from Chembridge Corporation, Budke *et al.* [225] reported a molecule able to inhibit RAD51 ssDNA binding, RI-1 (3-chloro-1-(3,4-dichlorophenyl)-4-morpholino-1H-pyrrole-2,5-dione). The mechanism of action of RI-1 was based on its chloromaleimide moiety, which binds covalently to the thiol group of the Cys319 residue on RAD51 surface by a Michael addition mechanism. This interaction was found to inhibit DNA binding (IC_50_ = 6.62 µM) and polymerization of RAD51 during nucleofilament formation. As the interaction site on RAD51 surface is highly conserved among mammalian, RI-1 bound also Rad51 from *Saccharomyces cerevisiae*, the Cys319 human RAD51 residue corresponding to its Cys377 residue, but not RecA where the site is absent. The Cys319 residue of RAD51 is located on the interface between two RAD51 monomers. This site overlaps the ATPase domain of RAD51, suggesting RI-1 may prevent nucleofilament formation and also modulate ATP binding. RI-1 treatment is associated with decreased RAD51 foci formation and increased γ-H2AX foci formation [226]. Importantly, RI-1 has been shown to produce an anti-cancer effect (IC_50_ range from 5 µM to 30 µM) when associated with mitomycin-C treatment in four cell lines (U2OS, HeLa, MCF-7 and SH2038).

A RI-1 analog, named RI-2 (1-(3,4-dichlorophenyl)-3-(4-methoxyphenyl)-4-morpholino-1H-pyrrole-2,5-dione), was developed to reduce probability of off-targets and to improve the stability of the molecule *in vivo* [227]. RI-2 lacks the chloromaleimide-based reactivity that potentially limits RI-1 use in animal models This compound was able to bind RAD51 at the same location as RI-1 does and inhibited DNA binding function of RAD51 (IC_50_ = 44.17 µM) in a reversible manner. As RI-1 (20 µM), RI-2 treatment (60 µM) led to HR inhibition in cells despite it lacks the Michael acceptor reactivity of RI-1.

### 7.5. IBR2 and IBR120

IBR2 is a small molecule able to inhibit RAD51 by mimicking the effect of BRC repeat binding to RAD51. BRC repeats are structural motifs that confer upon BRCA2 the property to bind to RAD51 [228]. IBR2 was discovered by screening a 24,000 molecules Nanosyn library using an inducible reverse yeast two-hybrid system to highlight molecules capable to inhibit BRC-RAD51 interaction [229]. IBR2 was shown to specifically bind to RAD51 to prevent its multimerization and therefore nucleofilament formation. Consistent with this, IBR2 diminishes the efficiency of HR and inhibits RAD51 foci formation in MCF-7 cells after IR treatment (20 µM for 8 h). IBR2-treated cells showed no effect of the molecule on RAD51 at the transcriptional level, but IBR2 was found to promote RAD51 degradation through the ubiquitin-proteasome pathway. IBR2 exhibited a potential therapeutic use for treating a broad spectrum of human malignancies, as it inhibited cell growth in various cancer cell lines, including K562, HeLa, MBA-MD-231, MBA-MD-435, MBA-MD-468, MCF-7, T47D and HBL100 (GI_50_ range from 11.5 to 16.0 µM). It was also tested *in vivo* as a monotherapy on a breast cancer xenograft model, leading to a decrease in tumour growth in nude mice. This same study also highlighted the promise of IBR2 as a treatment against hard-to-treat cancers, such as CML resistant to imatinib (a Bcr-abl inhibitor used to treat CML). For instance, IBR2 treatment of murine cells that express the imatinib-resistant mutant T315I Bcr-abl led to a decrease in cell proliferation and HR efficiency with associated apoptosis. Moreover, non-Obese Diabetic/Severe-Combined Deficiency mice injected with T315I cells and treated with IBR2 (100 mg/kg) showed a prolonged survival compared to mice treated with imatinib alone (125 mg/kg). Finally, co-treatment of K562 human leukemia cells, a BCR-ABL positive and chemo-resistant cell line derived from a CML patient, with IBR2 and imatinib showed an increase of apoptosis, suggesting a synergistic effect on cell killing.

Recently, IBR2 analogues were developed to improve the efficiency of the initial molecule [230]. All 24 newly synthetized analogues were screened for their ability to inhibit the growth of the triple-negative breast cancer cell line MBA-MD-468. Four of them exhibited at least a 1.6-fold greater effect than IBR2 (IC_50_ = 14.8 µM), especially the isoindolinyl derivative IBR120 (IC_50_ = 3.1 µM), which showed a 4.8-fold increase in growth inhibition activity compared to IBR2. IBR120 demonstrated anti-proliferative activity in many cancer cell lines (breast cancer cell lines K562, MCF-7, MBA-MD-231, MBA-MD-361, MBA-MD-435, MBA-MD-468, HsT578-T, T47D, human osteosarcoma cell line U2OS, human glioblastoma cell line T98G and human cervical cell line HeLa), with IC_50_ in the low micromolar range (from 3 to 5 µM for most tested cell lines). Using docking analysis, three RAD51 residues (V189, Y191 and Q206) were identified as potential hydrogen bonding sites for interaction with IBR120. As IBR2, IBR120 prevented RAD51 multimerization and decreased HR efficiency. IBR2 and IBR120 also share structural similarities, the central ring structure in IBR120 being only one carbon smaller than that of IBR2. In contrast to IBR2, the effects of IBR120 *in vivo* has not been yet investigated. This study provided interesting insights into the chemical groups and structures involved in IBR2 and IBR120 inhibition of RAD51, and suggested the possibility of improving IBR2 inhibitory activity on RAD51.

Although no clinical trial involving RAD51 inhibitors has been initiated, these molecules remain promising in light of a combinatory treatment as well as a potential therapy for hard-to-treat cancers such as CML.

## 8. The RAD52 Protein

*Rad52* was firstly identified in *Saccharomyces cerevisiae* by a genetic screen for mutants sensitive to IR treatment [231]. Absence of Rad52 confers important defects, Rad52 being involved in all HR pathways both Rad51-dependent (Double-Strand Break Repair and Synthesis-Dependent Strand Annealing) and Rad51-independent (Single-Strand Annealing) [7]. Human RAD52 is structurally and biochemically close to ScRad52, even if inactivation of RAD52 is not lethal in mice [232]. RAD52 binds ssDNA, promotes DNA annealing, interacts with RAD51 to modulate its DNA strand-exchange activity upon the reaction conditions and interacts with RPA [233,234]. Previous studies have shown that the N-terminal part of RAD52 is involved in the oligomeric ring formation leading to the RAD52-ssDNA binding. This interaction is due to a large number of positively charged residues in the groove of the RAD52 ring [235,236]. RAD52 also possesses a second DNA binding site that binds dsDNA [237]. Genome-wide study has lately characterized *RAD52* as a lung cancer susceptibility gene, the region containing *RAD52* gene was amplified in patients affected by lung cancer. The authors showed that overexpression of RAD52 causes an increase in cell proliferation and that depletion of Rad52 in mouse lung cancer cells leads to cell death [238]. Recent studies have highlighted importance of RAD52 in human cancer cell lines deficient in BRCA1, PALB2 [239] or BRCA2 [240], as its depletion led to cell death in a synthetic lethality manner. Thus, developing therapeutic strategies targeting RAD52 to treat PALB2, BRCA1 or BRCA2 mutant cancers might be considered. Currently, no molecules have been described to inhibit RAD52 whether it is by preventing RAD52 ring formation, dissociating RAD52 ring, preventing RAD52-DNA binding or modulating RAD52 post-transcriptional modifications. These approaches will need high-throughput screening of small molecules libraries in addition to biochemical and cellular assays to characterize and improve the effects of potential inhibitors. To this end, advances have been made in the characterization of RAD52 activity at both the biochemical and cellular levels [241,242].

## 9. The Non-Homologous End-Joining (NHEJ) Proteins

**Figure 3:**
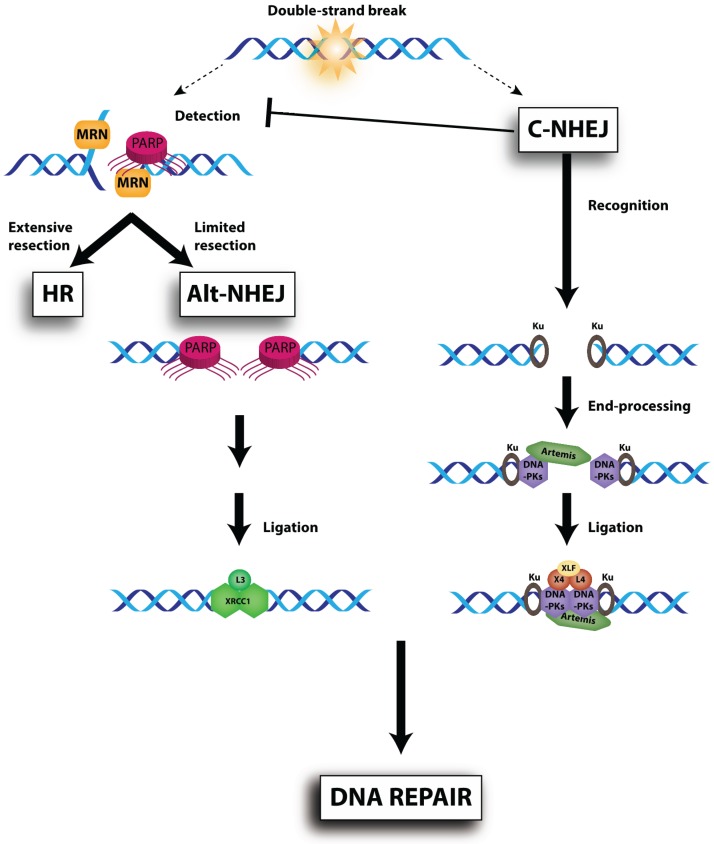
Schematic representation of DNA repair by non-homologous end-joining (NHEJ): classical NHEJ (C-NHEJ) or alternative NHEJ (alt-NHEJ).

Non-Homologous End-joining (NHEJ) is a major double-strand break repair pathway, accounting for 75% of DSB repair in proliferating cells [243]. This repair mechanism can operate throughout the cell cycle, but is suppressed by HR which functions S-G2 phases, when the sister chromatid is available as a repair template [2,244]. NHEJ can be divided into two distinct sub-pathways: classical NHEJ (C-NHEJ), the main canonical DNA-PK-dependent pathway, and alternative NHEJ (Alt-NHEJ) (Figure 3). A particularity of NHEJ is the polyvalence of the nuclease, polymerase, and ligase activities, which provides the ability to repair a broad spectrum of DNA-end substrate configurations that can possibly arise at a DSB (e.g., DNA ends of variable overhang length, sequence or chemistry) [245]. Hence, targeting this main repair pathway is one strategy to fight cancer by increasing cancer cell sensitivity to DSB-inducing agents.

C-NHEJ is composed of 3 steps: break recognition, end-processing and ligation. During C-NHEJ, the double-strand break is recognized by the Ku70/Ku80 complex, a dyad-symmetrical molecule that encircles duplex DNA with its ring structure. Ku recruitment and binding helps to stabilize NHEJ enzymes at the DNA termini. Indeed, Ku allows the recruitment of DNA-PKcs, the Artemis complex, the λ and μ polymerases, and a ligation complex composed of XLF (XRCC4-like factor, also called Cernunnos), XRCC4 and DNA ligase IV. The catalytic subunit DNA-PKcs serves as a platform for NHEJ repair. DNA-PKcs is a serine/threonine protein kinase which belongs to the PI3K (phosphatidylinositol 3-kinase) family. Several types of DNA ends activate DNA-PK, leading to its auto-phosphorylation and the downstream phosphorylation of Artemis, an exo- and endonuclease. As a result, DNA end-processing can occur to render the DSB extremities competent for ligation. The recruitment of the XLF-XRCC4-DNA ligase IV complex can then allow the direct ligation of the break ends. Unfortunately, this step implies the loss of base pairs and explains why NHEJ is not an entirely faithful mechanism. C-NHEJ is able to inhibit other DNA repair pathways. In the absence of this inhibition, PARP1 and the MRN (MRE11–RAD50–NBS1) complex are recruited to the DNA damage site. When resection at the break-ends is limited, the DSB can be repaired by the alt-NHEJ pathway, which implicates DNA ligase III and XRCC1. Alt-NHEJ functions as a backup repair pathway, detected only when C-NHEJ is compromised. In contrast to C-NHEJ, Alt-NHEJ is promoted by poly (ADP-ribose) polymerase-1 (PARP-1) and relies on the activities of distinct factors, such as X-ray cross complementing factor 1 (XRCC1), DNA ligase IIIα, polynucleotide kinase (PNK), and Flap endonuclease 1 (the latter two are not shown on the figure).

### 9.1. Selective DNA-PK Inhibitors

The DNA-PK holoenzyme is composed of a regulatory heterodimer (Ku70 and Ku80 subunits) and a 460 kDa catalytic component, DNA-PKcs. Given that DNA-PK is central to NHEJ, its loss results in inefficient repair of DSBs, increasing sensitivity of cells to ionizing radiation. Conversely, its overexpression can lead to resistance to ionizing radiation in tumour cells. Hence, the search for specific inhibitors against DNA-PK has rapidly gained interest and potential. To date, the most promising approach to DNA-PK inhibition has been to target the ATP-binding site of the kinase catalytic subunit DNA-PKcs using small inhibitors [246]. A major interest around these inhibitors lies in their radio- and chemosensitization capabilities. However, activity against other PI3K proteins and poor pharmacokinetic issues have brought some difficulties into the development of specific molecules targeting DNA-PK (depicted in Figure S6).

#### 9.1.1. Wortmannin

Wortmannin is a sterol-like fungal metabolite, and as mentioned previously, is a non-competitive general inhibitor of PI3K that has been used to target both DNA-PK and ATM. Wortmannin causes the irreversible alkylation of Lys802, which is located in the ATP-binding site of DNA-PKcs and critical for the phosphate transfer reaction [247]. However, the lack of specificity of this compound, its poor solubility, and *in vivo* toxicity have limited its clinical application [248].

#### 9.1.2. LY294002

LY294002, a morpholine-containing chemical compound, is also a non-specific DNA-PK inhibitor, most frequently used *in vitro* as a PI3K inhibitor [249]. It acts by reversibly binding to the kinase domain of DNA-PK in a competitive manner [248]. LY294002 has also been shown to inhibit Ras signalling pathway, rendering it a potential antitumour drug [250]. Unfortunately, *in vivo* toxicity has made its clinical use in humans unconceivable. Nonetheless, the basic chemical structure of LY294002 has been used as the basis for the development of more potent novel inhibitors.

#### 9.1.3. NU7026

NU7026 is a competitive and highly selective inhibitor of DNA-PK. Importantly, it shows no activity against ATM and ATR and has a 60-fold selectivity towards DNA-PK compared to PI3K [251]. It has been shown that NU7026 potentiates the cytotoxicity of topoisomerase II poisons used in the treatment of leukemia. [252]. NU7026 enhances the growth inhibition of chemotherapeutic agents such as idarubicin, daunorubicin, doxorubicin, etoposide, amsacrine, mitoxantrone but does not potentiate the effects of camptothecin or cytosine arabinoside. However, considering the pharmacokinetics of this compound, Nutley *et al.* suggested that sufficient exposure time and concentrations could not be achievable in patients for its therapeutic use in synergy with radiation or chemotherapy*.* Hence, in spite of its high selectivity, NU7026 requires optimization for clinical use [253]. Lately, NU7026 has been shown to enhance the cytotoxic effect of irradiation gastric cancer cells. This heightened cytotoxicity was explained by an increase in DSB formation, resulting in G2/M cell arrest and possibly higher levels of apoptosis [254].

#### 9.1.4. NU7441

NU7441 was designed from the LY294002 backbone with improved potency and similar promise to NU7026. In cultured cell lines, NU7441 showed strong inhibition of DNA-PK, caused persisting doxorubicin- and IR-induced DSBs, and resulted in a modest increase in HR activity [255]. NU7441 has also been shown to increase the cytotoxicity of IR and etoposide in SW620, LoVo, and V3-YAC colon cancer cells but not in DNA-PKcs-deficient V3 cells, showing the specificity of the inhibitor for DNA-PK [256]. In xenograft colon cancer models, NU7441 was maintained at concentrations necessary for chemopotentiation *in vitro* for at least 4 h at non-toxic doses. Finally, etoposide-induced tumour growth delay was increased two-fold without enhancing toxicity. NU7441 was further found to radiosensitize human leukaemic MOLT-4 cells, with the authors proposing that NU7441 could be a platform for the development of new radiosensitizers in radiotherapy [257]. More recently, it has been shown that NU7441 markedly sensitizes Hep3B cells to the anti-proliferative effects of harmine, a naturally occurring alkaloid with HR suppression ability that induces DSBs through the inhibition of RAD51 recruitment. Interestingly, this suggested that targeting NHEJ and HR could be used as a new synthetic lethal approach in anti-cancer treatment [258]. Furthermore, it has been shown that NU7441 (and NU7026) could dramatically enhance WAY-600 (a potent mTOR inhibitor) cytotoxic and pro-apoptotic effect against colorectal cancer cells [259].

#### 9.1.5. KU-0060648

KU-0060648 was investigated in a panel of human breast (MCF7, T47D and MDA-MB-231) and colon (LoVo and SW620) cancer cells. This compound inhibits cellular DNA-PK auto-phosphorylation and PI3K-mediated AKT phosphorylation [260]. Hence, KU-0060648 is a potent dual inhibitor of DNA-PK and PI3K, which inhibits cell growth and enhances the cytotoxicity of topoisomerase II poisons in a cell line-dependent manner. KU-0060648 showed good oral bioavailability and pharmacokinetics. Recently, a study showed that KU-0060648 (and NU7441 previously described*)* can improve the Cas9-mediated genome editing [261].

#### 9.1.6. ICOS Compounds

The ICOS Corporation (The name Icos comes from icosahedron, a twenty-sided polygon—a shape common to many viruses) small molecule library has been used to identify compounds inhibiting the DNA-PK-dependent phosphorylation of a p53 peptide substrate [262]. The first molecule identified was the moderately potent kinase inhibitor IC60211, an arylmorpholine. Optimization of this compound resulted in many DNA-PK selective inhibitors that maintained the arylmorpholine structure, none of which were cytotoxic at concentrations of <50 μM. IC86621, a methyl ketone derivative of IC60211, was not the most active compound. However, it was a suitable, easily synthesized, and chemically stable representative of this inhibitor class. IC86621 was structurally very simple and demonstrated no activity against a panel of distantly related protein kinases or against closely related protein kinases, including ATM and ATR. However, the drug has activity against the closely related PI3K. Surprisingly, IC86621 reduced spontaneous HR but also HR induced by the I-*Sce*I nuclease [263]. The compound did not induce fusions in Ku70 knock-out mouse cells, suggesting it requires the holoenzyme to be effective [264]. IC86621 inhibition of DNA-PKcs prevents chromosomal end protection through mechanism reminiscent of dominant-negative reduction in DNA-PKcs activity. [265].

IC87361 is the most highly evolved morpholino-flavonoid and is 50-fold more selective for DNA-PK than for the PI3K catalytic subunit p110β. At least 4 h exposure to a 10 μM concentration of IC87361 was required for radiosensitization. It presents a rapid clearance from circulation and low bioavailability which usually are the major barriers to the clinical use of these compounds [266]. IC486241 is an inhibitor studied by Davidson *et al.* in 2012. The authors showed that treatment of three breast cancer cell lines (MCF7, BT-20 and MDA-MB-436) with doxorubicin or cisplatin in combination with the DNA-PKcs inhibitor IC486241 synergized the cytotoxic effects of these drugs [267]. Davidson *et al.* showed that Irinotecan and IC486241 synergized in the killing of colon cancer cells [268].

#### 9.1.7. OK-1035

Kinetic studies have first indicated that OK-1035 was an inhibitor of DNA-PK activity in an ATP-competitive manner [269]. Functionally, OK-1035 has been shown to affect p53 activity in cells following DNA damage [270] and to inhibit DNA repair in radioresistant L5178Y cells [271]. However, no information concerning effects on PI3K-kinase activity or DSB repair was reported. Stockley *et al.* also found that OK-1035 is a weaker inhibitor of DNA-PK than initially reported [272]. Furthermore, this compound displayed weak pharmacokinetic properties, making it unsuitable for further clinical development.

#### 9.1.8. SU11752

SU11752 is an ATP-competitive drug that can cause inhibition of DNA-PK activity [273]. SU11752 was shown to lack the required potency for *in vivo* trials; hence, no studies have since been published using this particular inhibitor. Nonetheless, the authors suggested that SU11752 could be used to develop a new class of specific DNA-PK inhibitors.

#### 9.1.9. Vanillin

Several natural plant-derived compounds, including vanillin, cinnamaldehyde, coumarin, umbelliferone, anisaldehyde and tannic acid, are known to possess moderate antimutagenic properties. They also sensitize cancer cells to the lethal effects of DNA-damaging agents [274]. Particularly, the phenolic aldehyde vanillin, derived from a species of vanilla pods, has also been shown to inhibit DNA-PK. Following IR of human ovarian carcinoma cells, vanillin specifically affected NHEJ and was selective for DNA-PK over ATM and ATR [275]. Hence, through their specific action, vanillin-based molecules could be further used to investigate DNA-PK and NHEJ implication in DSB repair. Recently, vanillin was utilized to inhibit the aberrant activation of centrosomal DNA-PK in the absence of SF-1 (a tissue-specific transcription factor expressed mainly in the adrenal glands and gonads), which reversed a cascade of centrosomal events leading to genomic instability [276]. The potentially beneficial impact of vanillin-based molecules in the prevention of genomic instability and cancer could also be of interest.

#### 9.1.10. NK314

NK314 is characterized as an anti-tumour agent possessing activity against topoisomerase II alpha (TOPOII) [277,278,279]. Interestingly, its dual molecular targeting properties have first been discovered in 2011, Hisatomi *et al.* showed that NK314 could induce the degradation of DNA-PKcs in adult T-cell leukemia-lymphoma cell lines, causing impaired DNA DSB repair [280]. The mechanism by which this drug inhibits DNA-PK remains unclear. NK314 is currently in clinical trials for the treatment of ATL, due to its impressive activities in preclinical models, illustrating the potential for using DNA-PK inhibitors in the clinic.

#### 9.1.11. CC-115

CC-115 is an improved ATP-competitive molecule developed from the mTOR kinase inhibitor CC-223 [281]. CC-115 is hence a dual inhibitor for DNA-PKcs and mTOR and the first clinical trial are undergoing to assess its safety and action in patients with advanced solid tumours and hematologic malignancies that are unresponsive to standard therapies.

### 9.2. Nucleotide-Based Inhibitors of DNA-PK

This class of inhibitors is a secondary type of drugs that few have focused on, despite their potentially great clinical efficacy as DNA-PK inhibitors. Indeed, the biological nature of these molecules could allow them to overcome the poor solubility and short serum half-lives of inhibitors previously discussed.

#### GRN163L

GRN163L (Imetelstat), is a 13-mer oligonucleotide initially developed as a direct inhibitor of the telomerase active site. In 2013, Shawi and colleagues showed that GRN163L inhibits DNA-PK phosphorylation and increases H2AX phosphorylation following treatment with the nucleotide analog fludarabine in CLL lymphocytes [282]. Inhibition of DNA-PK by GRN163L has also been found to be equivalent that observed with the use of NU7026. Together these observations indicate that GRN 163L, in addition to inhibiting telomerase activity, enhances chemotherapy through DNA-PK inhibition and impaired DNA damage repair. Whether GRN163L can be efficiently used in synergy with other DNA damaging therapies remains to be shown. However, from this time, studies have been focused on its use as a telomerase inhibitor.

### 9.3. Ku70/Ku80 Inhibitors

It has been shown that Ku70/80 levels are increased in a number of tumours suggesting that tumour survival may rely on these proteins. Recently, it has been shown that up-regulation of Ku70 expression in renal carcinoma cell line (786-O) could inhibit cell apoptosis and reduce susceptibility to radiation. Conversely, deletion of Ku70 induces cell apoptosis and significantly enhanced the sensitivity to radiation [283]. These observations suggest that Ku70/Ku80 may be a potential target for inhibition in cancer therapy in the future, although to date, specific inhibitors against Ku70/Ku80 have not been identified.

### 9.4. DNA Ligase IV Inhibitors

DNA ligase IV is the ATP-dependent DNA ligase that catalyzes the ligation step in NHEJ. Inhibiting the activity of DNA ligase IV is viewed as another approach to increase the sensitization of cancer cells to DNA damage (Skeletal representation in Figure S7).

#### 9.4.1. L189

Chen and colleagues used a computer-aided drug design, from a database of 1.5 million commercially available low molecular weight chemicals, in order to identify compounds predicted to bind to a DNA binding pocket within the DNA binding domain of DNA ligase I, thereby inhibiting DNA end-joining. It was found that 10 of 192 candidates specifically inhibited purified human DNA ligase I with some affinity for DNA ligase III. Of interest, L189 used in *in vitro* DNA joining assays showed inhibitory activity against DNA ligase I, III, and IV. L189 increased the cytotoxicity of MMS and IR in three cancer cell lines (breast, cervical, and colon) but not in a normal breast epithelial cell line. Hence, this suggests that L189 could be used as a potential compound for the development of treatments [284]. However, *in vivo* data and clinical trials are required to confirm the efficacy of this inhibitor.

#### 9.4.2. SCR7

SCR7 is a L189 derivative that was identified as a specific inhibitor of DNA ligase IV [285]. SCR7 inhibits end-joining of DSB by blocking ligase IV-mediated joining. SCR7 interferes specifically with ligase IV DNA binding, but not that of T4 DNA ligase or ligase I. Through its ligase IV-dependent inhibition of NHEJ, SCR7 has been shown to activate the intrinsic apoptotic pathway in cells and disrupt tumour progression in mouse models. SCR7 can also significantly enhance sensitivity of DSB-inducing agents (IR and etoposide) administered in combination. This NHEJ inhibitor offers therefore a great strategy toward the treatment of cancer and improvement of existing therapeutic agents.

### 9.5. XRCC4 Inhibitors

XRCC4 may function as an additional NHEJ scaffolding protein, responsible for the recruitment of other NHEJ factors to the site of the damage. XRCC4, in complex with ligase IV, is implicated in the NHEJ ligation step.

#### Salvianolic Acid B, Lithospermic Acid, and 2-O-Feruloyl Tartaric Acid

In 2011, Sun *et al.* screened a traditional Chinese medicine (TCM) database, TCM Database@Taiwan, and have identified three XRCC4 potent inhibitors [286]. Salvianolic acid B, lithospermic acid, and 2-O-feruloyl tartaric acid all bind to the DNA ligase IV binding region on XRCC4 (structures depicted in Figure S7). As potential enhancers of radiotherapy, the use and efficacy of these inhibitors require further assessment *in vitro* and in *in vivo* models. To date, no inhibitor targeting XRCC4 has been described *in vivo* and in clinical trials.

### 9.6. Alt-NHEJ Inhibitors

In a recent analysis of clinical samples from chronic myeloid leukemia (CML) patients, 50% of bone marrow mononuclear cells from patients with imatinib-resistant disease, as well as all patients in blast crisis, presented elevated levels of 2 Alt-NHEJ proteins, DNA ligase IIIα and PARP1, that correlated with hypersensitivity to the combination of DNA ligase and PARP inhibitors [287]. This suggested that increased dependence upon Alt-NHEJ can be identified and targeted in a significant fraction of CML patients having acquired resistance to the first line therapy and exhausted treatment options available. The Alt-NHEJ targeting strategy is likely to extend to a wide range of solid tumours, as there is evidence that this abnormality in DSB repair may also occur in a significant fraction of cell lines derived from different solid tumours [284], in particular from breast cancer with acquired or intrinsic resistance to anti-estrogens [288].

## 10. Conclusions

Cancer remains one of the most lethal diseases worldwide. In order to advance the management of this disease, we must identify agents that improve survival rates. Several of the DDR inhibitors we reviewed here have demonstrated potential to specifically and effectively eliminate cancer cells by DSB damage potentiation. While some require further *in vitro* and *in vivo* testing, for instance RAD51 inhibitors, other have advanced to clinical trials or are on the verge to do so. Among these, PARP inhibitors have shown therapeutic merit whether it is as a monotherapy or in combination with cytotoxic agents, with olaparib being the furthest progressed into clinical development and recently FDA-approved for use in women with BRCA-associated ovarian cancer. Promise for PARP inhibitors as a single agent also extends beyond BRCA cancers, as they have shown to be effective in sporadic tumours bearing no germline BRCA mutations, including high-grade serous ovarian cancers and triple-negative breast cancers [289] as well as in tumours with defects in other HR proteins. For instance, Ataxia-telangiectasia mutated (ATM) is a protein which also shares a synthetic lethal interaction with PARP [290,291]. ATM is frequently altered or deleted in several types of human cancers [292,293,294,295], in particular in haematological cancers [296,297]. Recent pre-clinical [298,299] and clinical studies [300] explored the applicability of olaparib synthetic lethality to cancers with disrupted ATM protein expression, specifically gastric cancer and mantle cell lymphoma (MCL). All studies demonstrated the powerful effect of olaparib synthetic lethality in MCL [298] and gastric [299] cancer cells with improvement in patients overall survival. It was also shown that the combination of PARP and ATM inhibitors has potential against ATM-proficient gastric cancer with p53 inactivation. These findings raise the prospect of targeting other p53-deficient cancers with the combined use of ATM and PARP inhibitors, and the possibility of a predictive biomarker for PARP-1 inhibitor activity in cancers harbouring a p53 disruption. PARPi are also effective in cells depleted for RAD51, RPA, RAD54, NSB1, MRE11, ATR, ATM, Chk1, and Chk2 [160]. However, more studies are required to optimize current clinical settings and combinations in which to administer olaparib. Moreover, the implementation of PARP inhibitors faces considerable limitations in that not all BRCA1/2 breast cancer patients have shown a response to PARP inhibition [156] and resistance can occur [301], emphasizing the need for new therapeutic alternatives.

**Figure 4:**
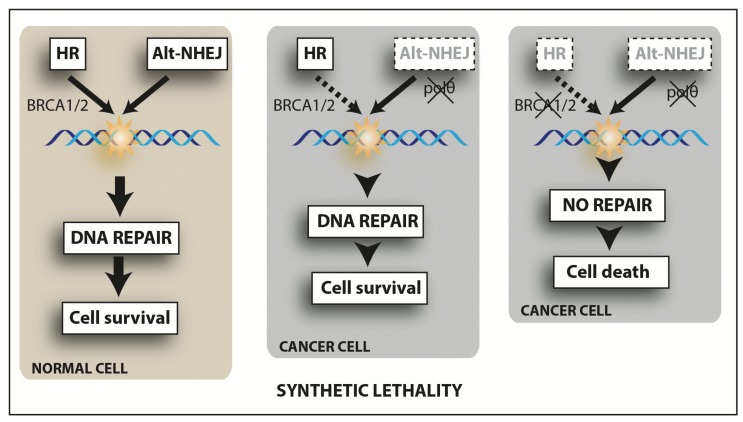
The synthetic lethality pathway model based on a deficiency in Alt-NHEJ and the double-strand break repair pathway.

The clinical development of NHEJ inhibitors and more specifically DNA-PKcs inhibitors falls behind, in comparison to the rapid clinical evaluation of PARP1 inhibitors. As mentioned above, a great number of agents with DNA-PKcs-inhibiting properties have been developed and pre-clinically characterized. Unfortunately, only CC-115 is currently being evaluated in a phase I trial (NCT01353625). The potential for combining DNA-PK activity with chemotherapeutics is clearly a strategy to cancer treatment. In particular, with the appropriate dose and delivery, DNA-PK inhibitors could be combined with HR inhibitors in a synthetic lethal strategy for cancer treatment. Recently, a novel synthetic lethal strategy was highlighted using Alt-NHEJ and HR (Figure 4). Depletion of polymerase theta and the BRCA genes has a synergistic effect on cell survival and represent a valid therapeutic avenue for tumours carrying mutations in homology-directed repair genes [302]. Altogether, several inhibitors described here have true potential for clinical use.

In normal cells, DSBs are repaired by homologous recombination or alternative non-homologous end joining (through polymerase theta). In cancer cells, one of these two repair pathways can be deficient. The inhibition of HR and polymerase theta leads to an accumulation of damage and cell death.

## Supplementary Files

Supplementary File 1
