# Supplementary material for: DNA Damage Signalling and Repair Inhibitors: The Long-Sought-After Achilles’ Heel of Cancer
Source: Biomolecules. 2015 Nov 20;5(4):3204–59. doi: 10.3390/biom5043204 (PMC4693276; doi:10.3390/biom5043204)
Supplement: Supplementary File 1 [file biomolecules-05-03204-s001.pdf]

Supplementary Material

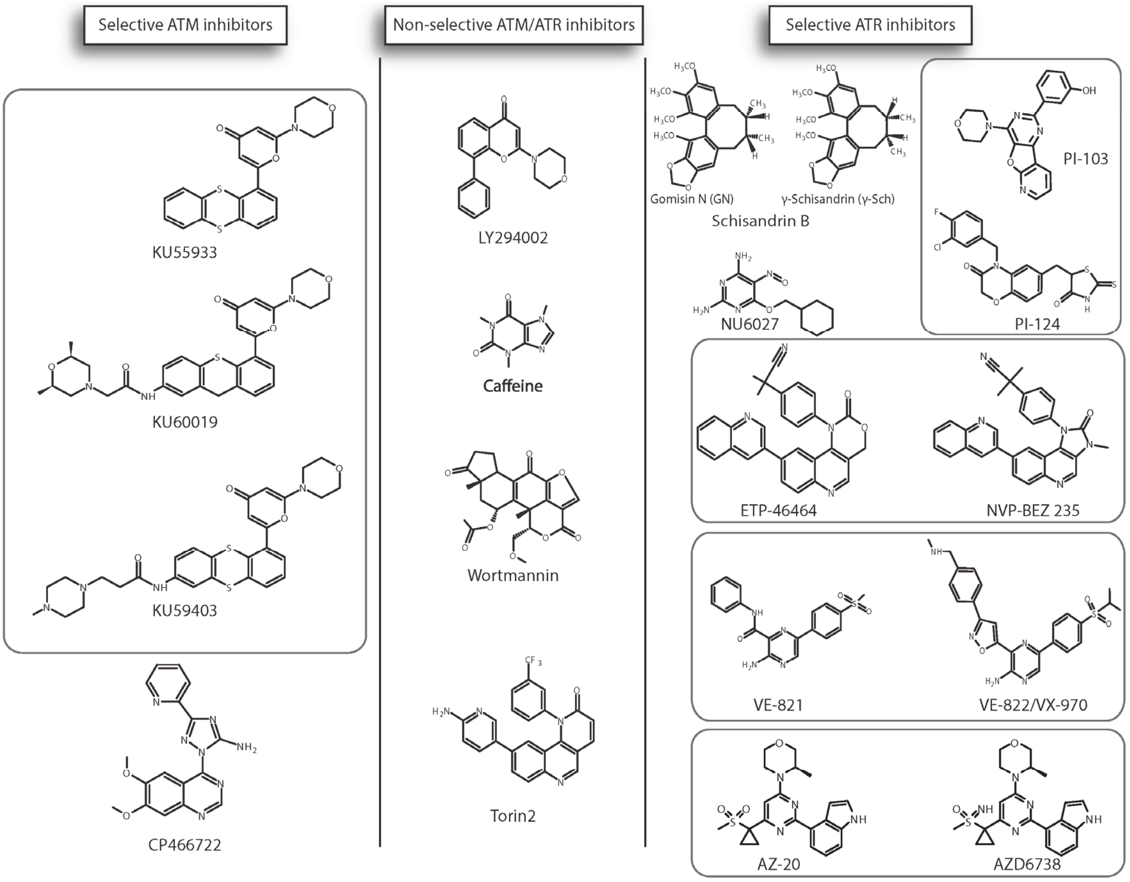

Figure S1. Selective ATM inhibitors (**left**), non-selective ATM/ATR inhibitors (**center**) and selective ATR inhibitors (**right**). Skeletal representation of the inhibitors. Grey boxes designate molecules sharing similarities.

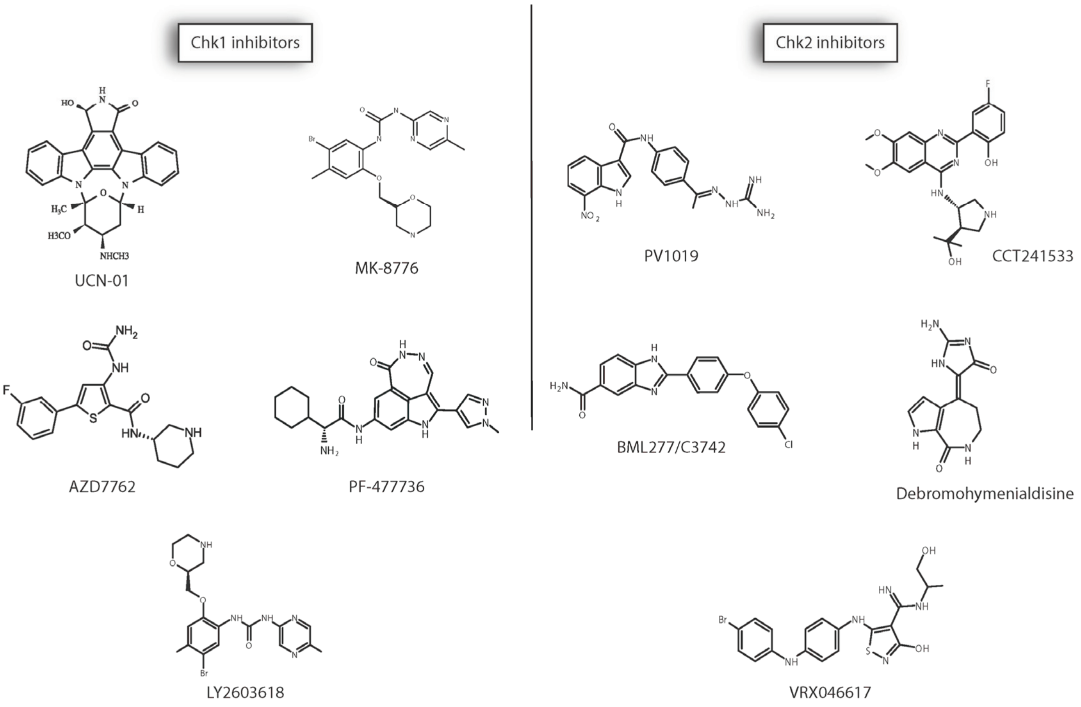

Figure S2. Chk1 inhibitors (**left**) and Chk2 inhibitors (**right**). Skeletal representation of inhibitors.

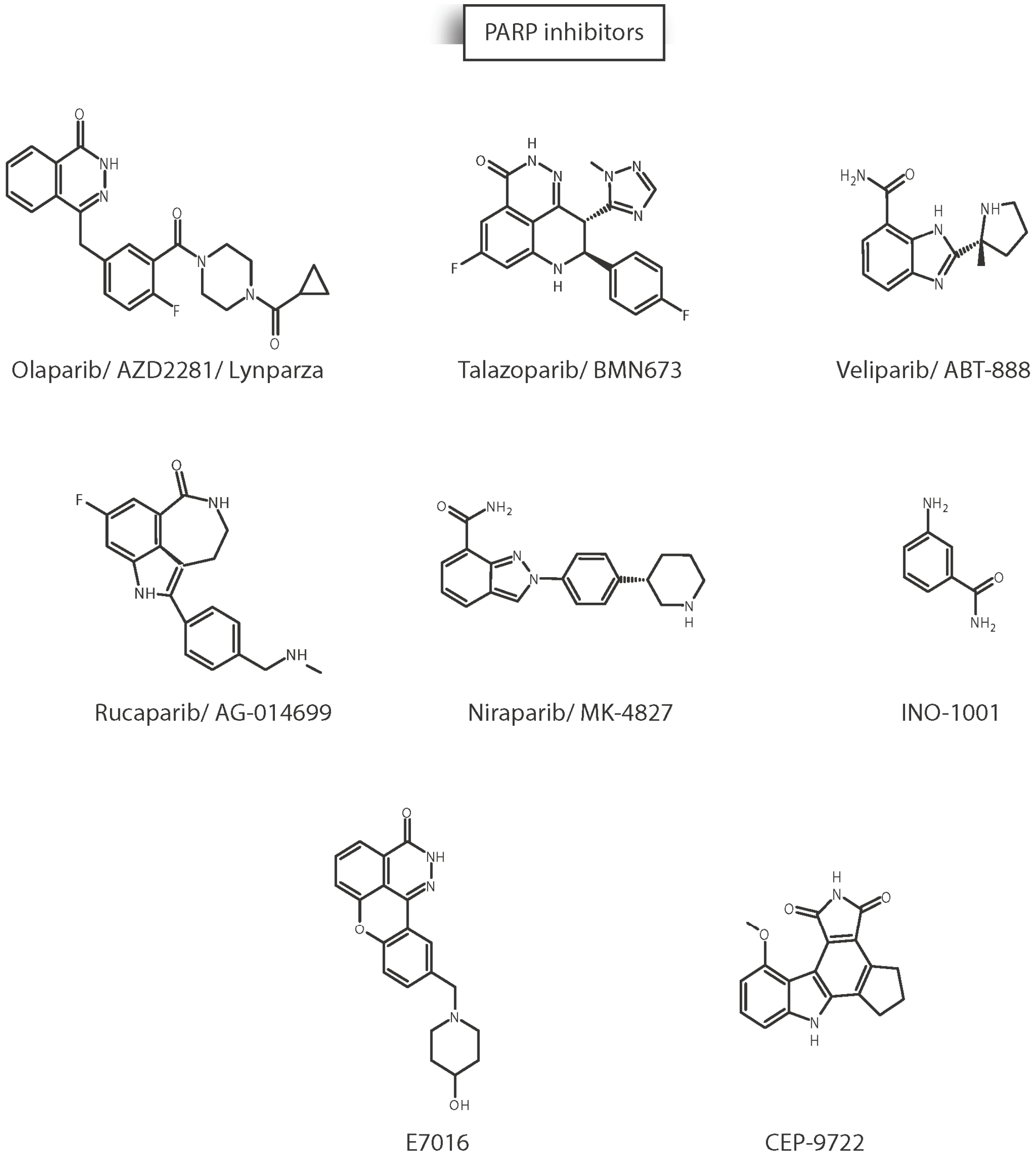

Figure S3. PARP inhibitors that have reached clinical evaluation.

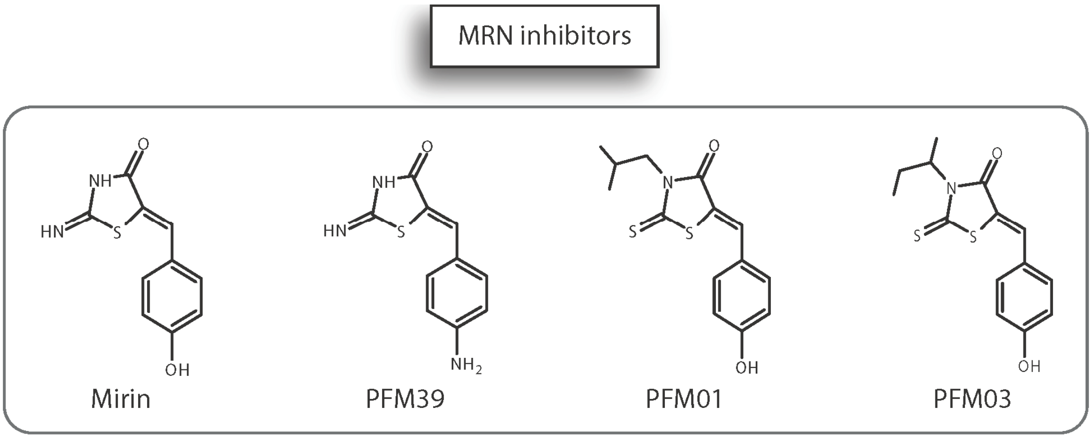

Figure S4. MRN (MRE11-RAD50-NBS1) complex inhibitors. Skeletal representation of inhibitors. The grey box designates molecules sharing similarities.

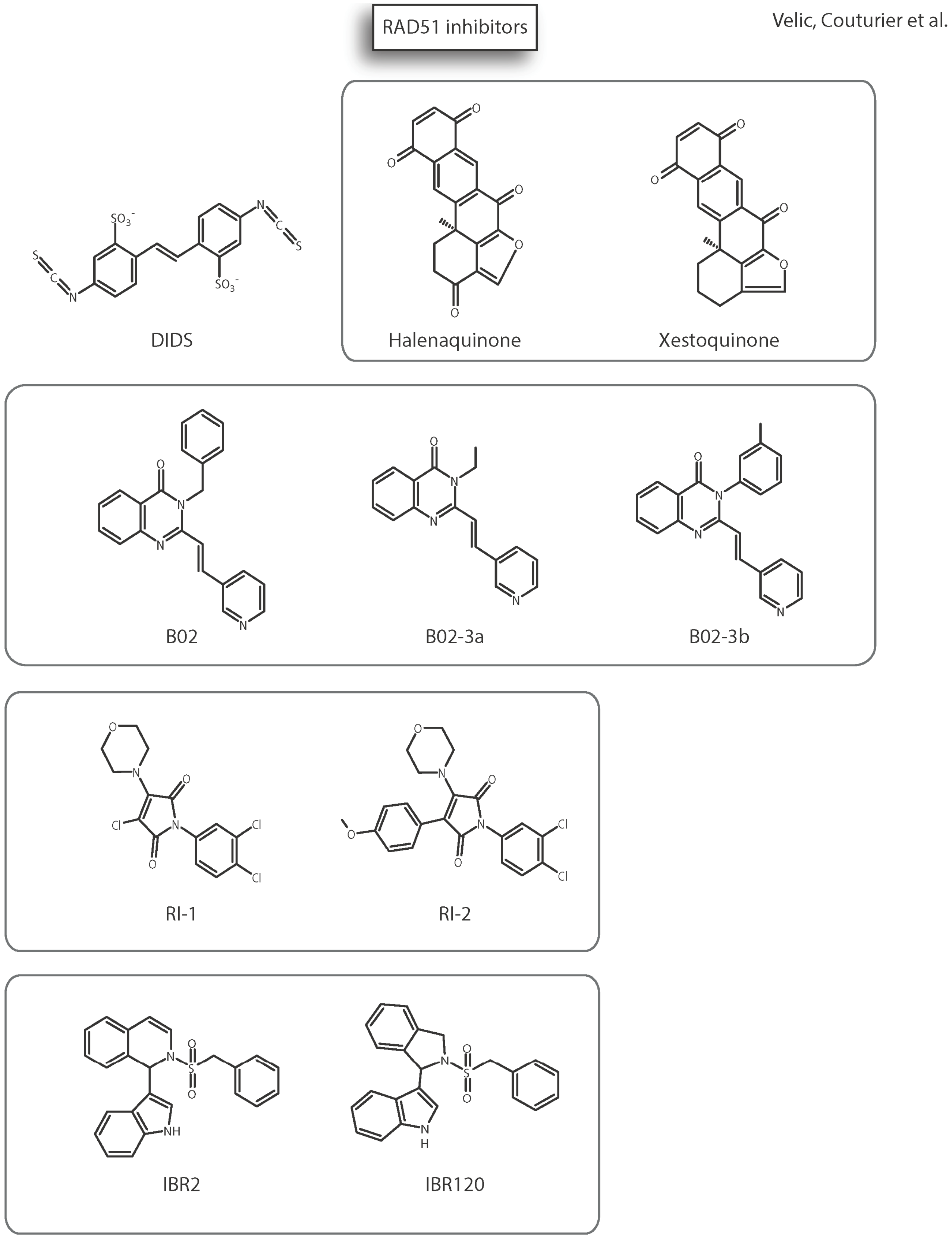

Figure S5. Skeletal representation of RAD51 inhibitors. Grey boxes designate molecules sharing similarities.

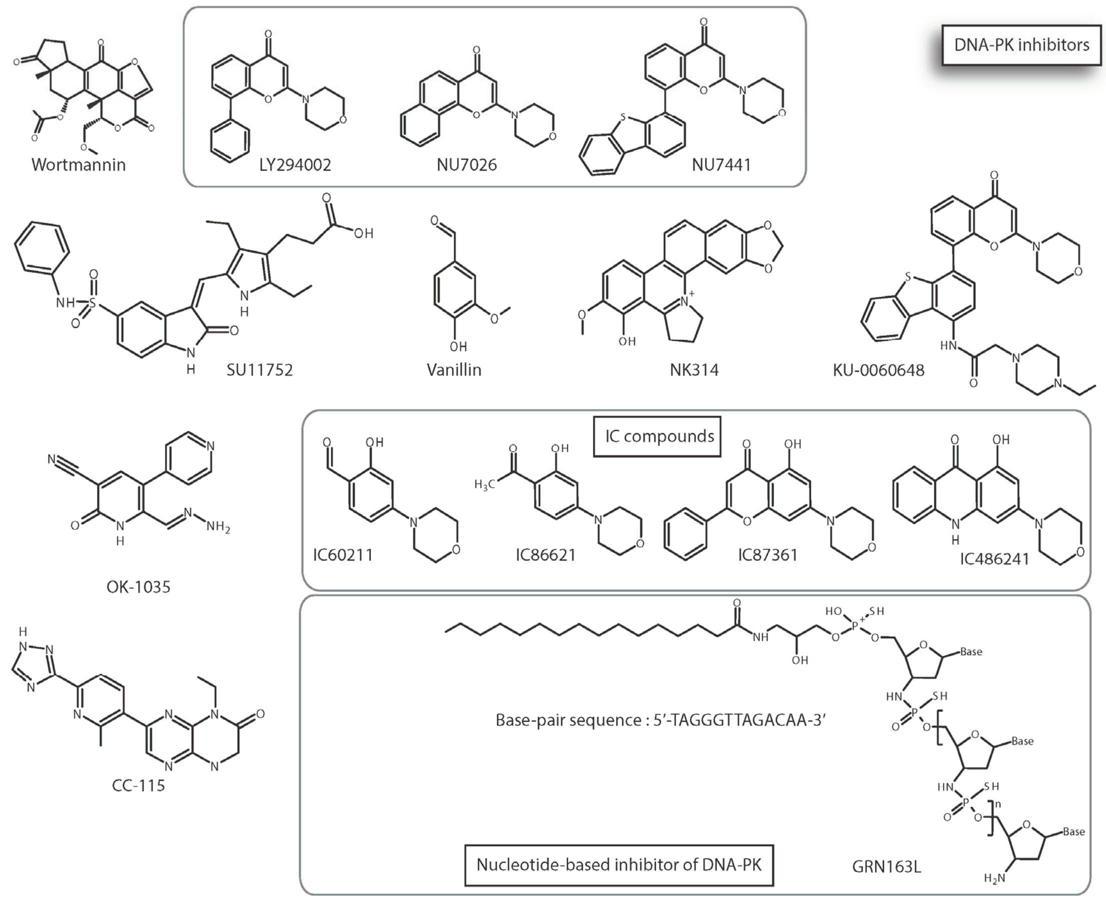

Figure S6. Skeletal representation of DNA-PK inhibitors. Grey boxes designate molecules sharing similarities.

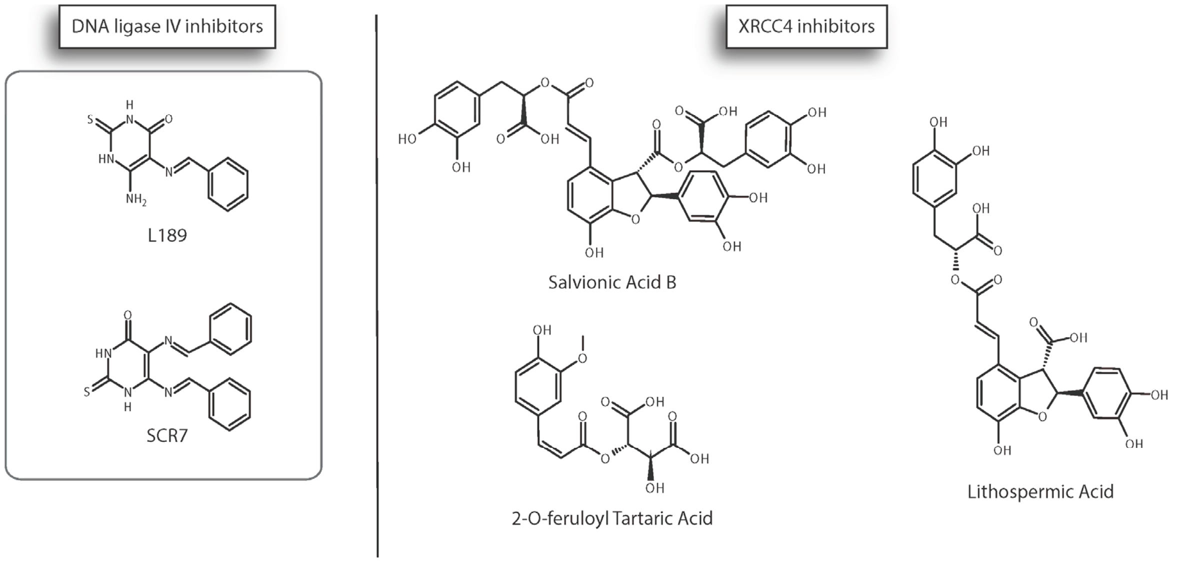

Figure S7. DNA ligase IV inhibitors (**left**) and XRCC4 inhibitors (**right**). Skeletal representation of inhibitors. Grey boxes designate molecules sharing similarities.

© 2015 by the authors; licensee MDPI, Basel, Switzerland. This article is an open access article distributed under the terms and conditions of the Creative Commons Attribution license (http://creativecommons.org/licenses/by/4.0/).
